# Opportunities and challenges for decarbonised, adaptable and resilient transport systems in the United Kingdom

**DOI:** 10.1038/s44333-026-00131-2

**Published:** 2026-11-02

**Authors:** Manu Sasidharan, Peter Helm, Omar Abo Madyan, Sam Cocking, Sripriya Rengaraju, David Alvarez Castro, Christos Vlachakis, Abdullah Kahraman, Ning Pan, Sungin Cho, Meg Bartholomew, Mashael Kamran, Jennifer Schooling, Ajith Kumar Parlikad, Abir Al-Tabbaa, Philip Greening, Roberto Palacin, Kristen Macaskill, Stephanie Glendinning, Phil Blyth

**Affiliations:** 1 Civil, Environmental and Geomatic Engineering Department, University College London, London, UK; 2 Department of Engineering, University of Cambridge, Cambridge, UK; 3 School of Engineering, Newcastle University, Newcastle upon Tyne, UK; 4 Edinburgh Business School, Heriott Watt University, Edinburgh, UK; 5 School of Engineering and the Built Environment, Anglia Ruskin University, Cambridge, UK

**Keywords:** Policy, Engineering

## Abstract

The transport sector faces complex challenges, including decarbonisation, resilience-building, technology integration, and alignment with evolving climate science. This study reviews over 120 documents outlining policies, programmes, and roadmaps that shape the UK’s transport strategies at national, regional, and local levels. National plans focus on zero-emission vehicles, autonomous systems, and digital technologies, emphasizing net-zero emissions through clean technologies, expanded electric vehicle infrastructure, and promotion of active travel. However, local strategies often operate in isolation, prioritizing net-zero targets while neglecting the impacts of climate change on infrastructure resilience and deterioration. Our analysis, supported by stakeholder workshops, reveals significant gaps in resilience standards, metrics, and statutory targets that undermine effective asset management. To address these deficiencies, we propose a conceptual framework integrating resilience and sustainability into transport planning. This framework balances new developments with proactive management of existing assets, effectively guiding future strategies to enhance the overall adaptability of the UK’s transport sector.

## Introduction

Transport plays a key role in shaping the societal fabric, extending far beyond its basic function of enabling movement. It fundamentally shapes the character of towns, cities, and rural areas, influencing living standards, public health, and overall quality of life. Transport’s effects can be transformative, either enhancing these aspects or detrimentally shaping them, depending on how transport systems are planned, developed, and managed. Improved connectivity and accessibility deepen labour markets, helping individuals access jobs and education, while well-functioning transport networks reduce direct costs for both individuals and businesses by enabling smoother flows of people and goods. Yet, current transport planning in the UK often overlooks the integrated management of decarbonisation, resilience, and sustainable asset strategies, leading to gaps that threaten long-term infrastructure reliability and societal prosperity. This paper synthesises key transportation strategies across local to national levels, revealing critical gaps between net-zero ambitions and infrastructure resilience, and proposes an integrated framework to future-proof the UK’s transport network.

The UK passenger transport system remains dominated by car use, with private vehicles accounting for the majority of trips and distances travelled across England, Scotland, Wales, and Northern Ireland. Recent national statistics indicate that cars were used for 60% of trips in England^1^, with similar proportions in Scotland (66%;^2^) and Northern Ireland (69%;^3^). In Wales, where recent comprehensive modal share data is lacking, commuting patterns show even higher car dependency at 79%^4^. Public transport, including buses and rail, accounts for a relatively modest share, comprising just 9% of trips and 15% of distances travelled in England^5^^,6^. Although usage varies regionally, recovery trends continue to be influenced by pandemic-related disruptions^4,7,8^. Bus usage outside London has improved since COVID-19 but has not yet reached pre-pandemic levels, remaining 46% below figures from 2002^9–11^. Rail usage showed consistent growth before the pandemic, with trips per person per year increasing by 61% from 2002 to 2019. Although rail travel also suffered declines during COVID-19, it recovered by 2023, returning to 2011 levels (18 trips per person per year;^10,11^).

Walking represents a significant proportion of short trips (29% in England) yet covers only 4% of total distances, reflecting that most walking trips (94%) are shorter than two miles^6,12^. Cycling remains minimal, accounting for only 2% of trips and 1% of distances^6^. Despite temporary increases during the pandemic and initiatives like cycling-to-work schemes^13^, overall cycling rates have shown limited sustained growth^11^.

In addition to passenger transport, significant volumes of freight are moved within the UK. Between 2012 and 2022, UK airports handled approximately 25.93 million tonnes (Mt) of freight annually, with the majority being international cargo^14^. Over the same period, rail transported about 90.6 Mt of freight annually^15^, while UK ports handled 479.92 Mt of goods per year^16^. Road transport via heavy goods vehicles (HGV) carries the largest share, moving around 1600 Mt of freight per year, with approximately 9% involving intermodal journeys^17^.

Changing travel patterns, particularly in urban environments^18^, are reshaping how people and freight move^19^. However, the UK’s transport infrastructure and services face significant challenges related to resilience, decarbonisation, and equitable accessibility. Each transport mode encounters various natural and anthropogenic risks, influenced by geographic location and engineering characteristics. For example, by 2080, around one-third of the UK railway network is projected to be susceptible to flooding, with anticipated increases in winter river flows placing one in twenty bridges at high risk of structural failure^20^. This vulnerability is exacerbated by chronic underfunding in maintaining ageing critical infrastructure^21^. More than half of the local road network in England and Wales currently has a remaining structural life of less than 15 years, with repair backlogs estimated at £16.3 billion^22^. Key disruptive mechanisms documented include:

- Short-duration, high-intensity extreme weather events such as intense rainfall, severe storms and wind events, extreme heatwaves, etc.^23^.
- Long-term climatic changes affecting weather patterns and extremes such as increasing average temperatures, changes in precipitation patterns, rising sea levels, increased frequency and intensity of storms^24,25^.
- Inadequate maintenance of infrastructure assets^21,26^.
- Changes in transport demand driven by social factors^27^, demographic shifts^28^ and economic developments^29^.
- Technological advancements and disruptions^29,30^.
- Security threats, including physical and cyber risks^31^.

The UK transport sector must also urgently implement measures to reduce their greenhouse gas (GHG) emissions. These actions are critical to fulfilling the commitments outlined in the Paris Agreement^32^ and achieving the targets set within the 2030 Agenda for Sustainable Development^33^. The transport sector remains the largest source of carbon emissions in the UK, accounting for 109 MtCO_2_ (26% of total CO_2_ emissions) in 2021, with approximately 91% attributed to road transport^34^. The primary contributors to these emissions were cars and taxis, which constituted 52% of domestic transport emissions, followed by HGVs at 20%, and vans at 17%. Another, often overlooked contributor to carbon emissions in the transport sector is the construction and maintenance of the associated infrastructure. The production of materials like steel and concrete is highly energy-intensive, leading to substantial global CO_2_ emissions of 3.7 Gt/yr and 2.6 Gt/yr, respectively [Data for 2023:^35^]. Additionally, maintenance activities often involve heavy machinery and processes that further contribute to the carbon footprint.

A decarbonised, adaptable, and resilient transport system is one that can maintain and adjust to acceptable performance levels in the face of increasing climatic, operational, and societal stresses, while also fulfilling a net zero agenda^36,37^. Given the complexity and interdependence of these issues, a holistic approach is necessary to transform existing transport networks into sustainable systems capable of adapting to a rapidly changing environment. This transformation must prioritise decarbonisation, incorporate resilience measures guided by up-to-date climate science, thoughtfully integrate advanced technologies while remaining attentive to potential additional risks, and comprehensively address health and socioeconomic impacts. In response, this study pursues the following research questions:

- How are resilience, decarbonisation, digitalisation, and climate science currently reflected in UK transport strategies?
- What challenges, gaps, and opportunities emerge from the perspectives of key stakeholders engaged in the UK transport sector?

To this end, this study provides the first comprehensive review of 124 publicly available strategies, policies, processes, and roadmaps developed at national, regional, and local levels across the UK, aimed at creating a decarbonised, adaptable, and resilient transport system (see ‘Methods’). The documents included in the review are listed in Table S1. Findings from this review are synthesised in the Results section, while key challenges and opportunities have been collaboratively identified through engagement with approximately 100 stakeholders (see Views from stakeholders section). This structured approach establishes the basis for developing a holistic framework for planning, designing, and managing future transport systems. The implications of this research are further examined in the Discussion, with conclusions presented in Section 5.

## Results

The findings from the review are discussed across four core themes, namely (i) building resilience to deal with an uncertain future, (ii) achieving decarbonisation, (iii) digitalisation and technology, and (iv) integrating updated climate science. While these themes are common across the transport sector, their treatment varies considerably across governance levels. National strategies tend to emphasise broad targets, standards, and inter-sectoral coordination, whereas local strategies often prioritise implementation challenges, funding constraints, and community engagement.

### Building resilience to deal with an uncertain future

To ensure that transport systems can continue to function effectively in the face of both present and future changes and disruptions, they must demonstrate adaptability and resilience to a wide range of hazards, events, and processes. These challenges can lead to increased service demands, infrastructure deterioration, or outright failure, and can operate over varying timescales, ultimately causing disruptions to transport networks and a reduction in overall performance^38–40^. This study evaluates the strategies and processes employed by UK transport authorities to anticipate potential disruptions or shocks to their infrastructure and operations, to resist and absorb these shocks, minimise their impacts, and ensure effective recovery. Furthermore, the review examines the capacity of these authorities to assess their vulnerability and preparedness in the face of such shocks, as well as their ability to adapt and transform their infrastructure over time to enhance resilience. Particular emphasis is placed on the risks associated with cascading failures, organisational perspectives regarding the importance of resilience, and evidence of practices such as stress testing and the development of resilience standards.

The National Infrastructure Commission (NIC) introduced a framework for assessing the resilience and adaptive capacity of the UK’s critical infrastructure systems and organisations^27^. In broad terms, the NIC^36^ adopts the definition of resilience provided by the United Nations Office for Disaster Risk Reduction (UNDRR, formerly UNISDR), as outlined in the Sendai Framework^37^: ‘the ability of a system, community, or society exposed to hazards to resist, absorb, accommodate, adapt to, transform, and recover from the effects of a hazard in a timely and efficient manner.’ This definition builds upon earlier work by UNISDR^41^. The terminology surrounding resilience is highly variable, encompassing a wide range of definitions and related terms^42^. For the task in hand, we adopt the resilience terminology defined within the NIC^27^ framework, as summarised in Table 1.

**Table 1:** Definitions of the various aspects of resilience and resilience-related terminology based on that adopted by the NIC^27,36^ or more broadly^42^

Terminology	Definition
Aspects of resilience	
Anticipate	Advance preparations to allow response to potential shocks and disruptions.
Resist	Advance preparations to allow a system to withstand or endure shocks and disruptions to prevent impact on infrastructure services.
Absorb	Actions taken in the event that there has been a negative impact on infrastructure services, that aim to minimise the effects.
Recover	Actions allowing the rapid restoration of service following a disruption.
Adapt	Actions that modify a system to enable continued service provision in the event of disruption or change.
Transform	Actions that alter the system to improve overall infrastructure performance.
**Other resilience related terminology used herein**	
Performance	The extent to which a system can undertake its designated function.
Hazard	An event that may act to apply pressure, or cause damage to an infrastructure system.
Shock	Pressure applied to an infrastructure system by a sudden (acute), unexpected event.
Stress	A longer term (chronic) pressure that acts to drive a system towards or beyond its normal working limits.

**Fig. 1:**
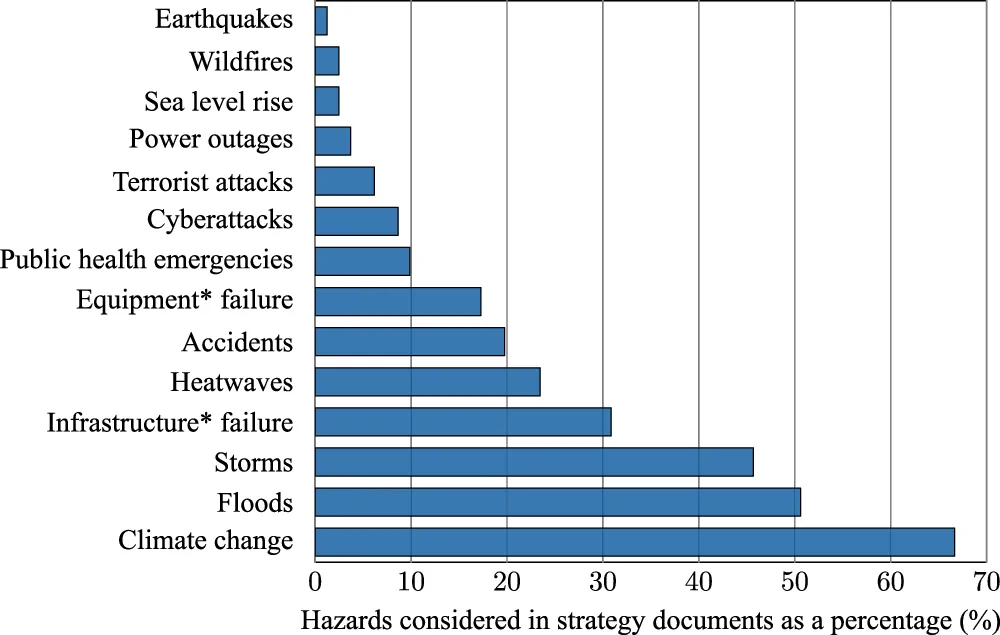
Differing hazards affecting the transport sector considered within reviewed strategies. *The review documents tended to differentiate between the civil infrastructure and mechanical, electrical and electronic equipment so that distinction was maintained herein.

Approximately one-third of the strategies reviewed do not explicitly address or anticipate the hazards posed by climate change. Furthermore, only half of the reviewed documents provide any substantial discussion of climate-related hazards, primarily focusing on floods (50%), storms (defined herein as heavy rainfall or snow, usually associated with high windspeeds, 46%), and heatwaves (23%), in descending order of perceived importance (see Fig. 1). In those documents that do not explicitly address climate change hazards, climate change is often discussed solely in the context of its link to CO_2_ emissions and as a rationale for the net-zero transition. This is influenced by the existence of statutory CO_2_ emission reduction targets, as established by UK law^43^. However, such an approach may underestimate the potential severity of disruptions resulting from climate change and related extreme weather events, indicating opportunities for improvement in resilience planning to ensure comprehensive preparedness.

**Fig. 2:**
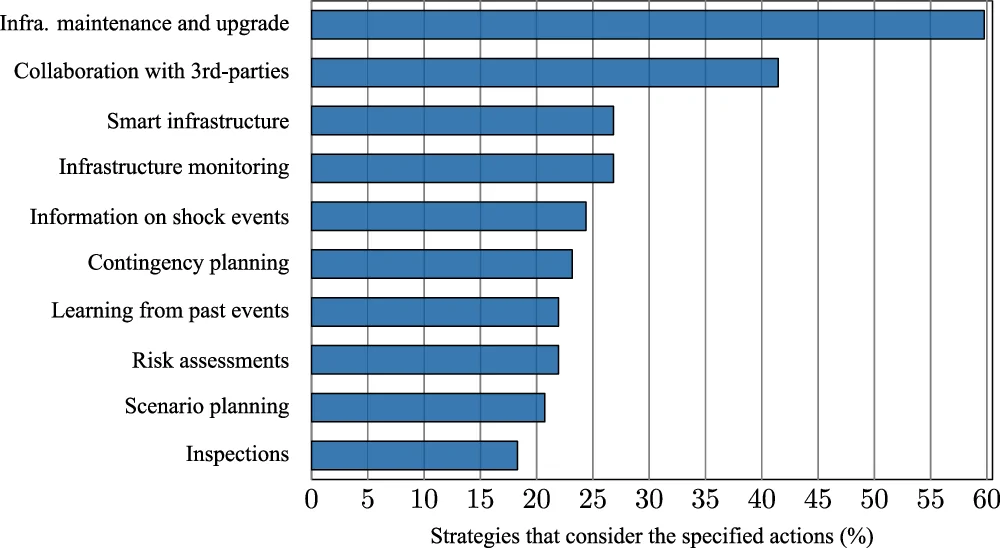
Plans within reviewed transport strategies for improving resilience and adaptability.

**Fig. 3:**
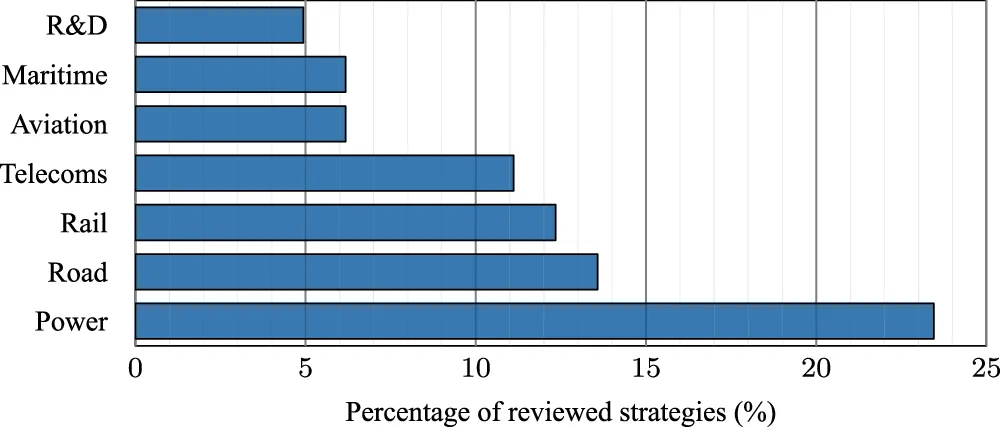
Strategies that consider dependencies with other sectors.

Collaboration with external organisations, such as weather agencies, to anticipate and prepare for weather-related disruptions is predominantly emphasised at the national level. This includes entities such as Network Rail, National Highways, Transport Scotland, the Welsh Government, and the Department of Infrastructure in Northern Ireland. However, such collaboration is rarely considered at the local level. A limited number of the reviewed documents, such as the Local Transport Plans for Cornwall, Devon, East Riding of Yorkshire, Glasgow, and Hampshire, as well as the Climate Change Adaptation Plans from Trinity House and the Northern Lighthouse Board, commendably acknowledged the need to enhance capacity to resist and absorb shocks to transport operations (See Fig. 2). However, the majority of other strategy documents reviewed, lacked explicit plans or actionable practices to address this issue. Also of note, the majority of the strategies reviewed failed to account for the interdependencies of their infrastructure with other critical systems, such as the power network (see Fig. 3). This is reflected in the low proportion of strategies that identify power outages as a potential hazard (see Fig. 1), despite the significant emphasis on electrification as a key pathway to achieving decarbonisation targets. Finally, the low proportion of strategies recommending inspection as a method for improving resilience is likely due to the fact that it is already a routine part the organisations’ asset management strategies.

The NIC framework^27^ highlights absorption and recovery as critical components of resilience, focusing on responses that occur after a failure or shock has impacted the network. However, the majority of the strategies reviewed place greater emphasis on anticipating, resisting, and adapting to potential hazards, with contingency planning remaining a relatively limited approach (see Fig. 2). Long-term resilience planning is predominantly concentrated at the national (e.g. Network Rail; the Civil Aviation Authority, CAA) and regional (e.g. Transport for South East) levels, with limited emphasis on local-scale implementation (e.g. East Renfrewshire Council and Herefordshire Council). Even where long-term resilience plans exist, there is an absence of established national resilience standards, and it remains unclear whether authorities have developed internal processes to address resilience issues. This gap in resilience standards also appears to affect the short-term/responsive management of disruptions, potentially highlighting a critical weakness in comprehensive resilience planning across all governance levels. Furthermore, a decision-making framework that can be followed in the event of a shock is not explicitly described, potentially highlighting a critical area where the wider transport sector lacks preparedness, increasing the risk of prolonged service outages and infrastructure damage.

The inherent uncertainties in forecasting the magnitude and frequency of future hazards, coupled with the complexities of designing and managing infrastructure to effectively resist and absorb their impacts, suggest that significant disruptions are likely to become increasingly frequent. As a result, the capacity to recover effectively from such events is anticipated to become a critical component of resilience strategies. A number of the challenges faced by differing transport sectors and modes, along with efforts made to address them is summarised in the following list.

- The *rail* sector is particularly susceptible to extreme weather events such as flooding and heatwaves. Existing adaptation measures include improvements to drainage systems, track reinforcement, and the use of heat-resistant materials. Recent region-specific initiatives have sought to develop tailored responses to local environmental challenges. However, while climate change and extreme weather are often acknowledged as sources of potential disruption and individual asset management groups may have well developed protocols to address them[for example, Network Rail’s Earthworks Technical Strategy:^44^], they tend to be compartmentalised as per the example here of earthworks and are not considered in the context of related infrastructure systems, furthermore the sector lacks specific resilience standards and formalised frameworks to guide infrastructure operators in decision-making and response during disruptive events.
- The *road* network faces considerable challenges, including disruptions caused by flooding, snow, and heat damage. Adaptation efforts have focused on improving road surfaces, enhancing drainage systems, implementing robust maintenance schedules^45^ and developing tools to understand deterioration of their asset portfolio over the long term as influenced by climate change [e.g. earthworks:^46^]. While nearly 45% of road-related strategies acknowledge the impacts of climate change and public health emergencies, many lack a comprehensive assessment of network resilience. Furthermore, clearly defined procedures for managing such shocks are often absent in these strategies.
- *Active travel* modes, including walking and cycling, are vulnerable to disruptions such as urban flooding and heat stress. Adaptation measures have centred on the development of green infrastructure, improvements to urban drainage, and the provision of shaded pathways^47^. Although the reviewed strategies often highlight the resilience benefits of sustainable urban planning, specific and consistent resilience standards for active travel infrastructure remain largely absent.
- The *maritime* sector faces significant risks from sea-level rise, storm surges, and extreme weather events. Adaptation efforts in this sector include strengthening port infrastructure, enhancing early warning systems, and improving emergency response capabilities. While maritime strategies frequently address these disruptions and aim to prepare for a wide range of risks—such as storms, power outages, floods, and cyber-attacks—many lack explicit decision-making frameworks. Notably, the Northern Lighthouse Board^48^ has adopted a whole-system approach, utilising data analytics to enable proactive maintenance and repair for the marine-navigation assets. The sector also recognises its critical interdependencies with other infrastructure systems, including power, telecommunications, road, and rail. Additionally, the Department for Transport’s (DfT) Maritime 2050 strategy^49^ seeks to establish incident precursors for the maritime sector, drawing inspiration from the approach employed by the Rail Safety and Standards Board (RSSB) for railways.
- The *aviation* sector faces diverse disruptions, including extreme weather events and volcanic ash clouds. Adaptation measures outlined by the CAA^50^ include enhancing runway durability, improving air traffic management, and developing contingency plans. While two of the UK’s largest airports have developed resilience plans, others are merely encouraged to develop their own. Although climate change and extreme weather are frequently acknowledged, explicit resilience standards and well-defined decision-making processes are often implied rather than clearly detailed. To address these shortcomings, the DfT and CAA have established the Aviation Resilience Planning Strategic Risk Group^51^, tasked with identifying critical interventions to enhance sector resilience and mitigate flight disruptions.

The findings from the review highlight the pressing need across all transport sectors for the development of formal resilience standards, comprehensive risk assessment frameworks, and well-defined decision-making procedures. The inherent interdependency of differing systems also strongly suggests that it is necessary to take a systems approach to resilience. Bridging these gaps is crucial for ensuring that the UK’s transport infrastructure is adequately equipped to withstand, adapt to, and recover from the challenges associated with climate change and other potential disruptions.

### Achieving decarbonisation in the transport sector

**Fig. 4:**
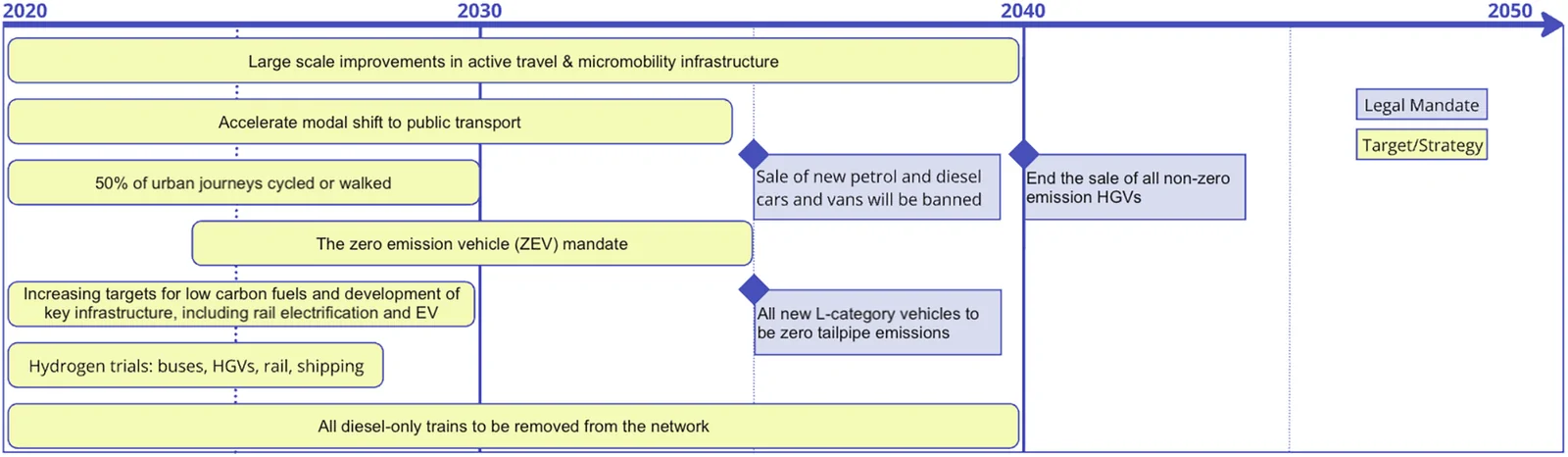
UK Transportation decarbonisation timeline highlighting key strategies and legal mandates.

The UK is committed to decarbonising the transport sector by 2050 through a series of key policies, investments and strategies focusing on transitioning to zero-emission vehicles, increasing active travel, and decarbonising freight and public transport (Fig. 4)^52–55^. A critical part of this will require the acceleration of modal shifts to public and active transport, making them more accessible, through improving infrastructure and encouraging the use of public transport. To support the ambitious target of ensuring that half of all urban journeys are undertaken by walking or cycling by 2030, a £2 billion investment has been allocated to enhance active travel infrastructure in towns and cities^56^. Equally, rural areas will require improved connectivity and upgraded infrastructure to ensure that such efforts are effectively implemented across the entire country. Short journeys, particularly those under five miles, which accounted for 58% of private car trips in 2019, offer significant potential for emission reductions through active travel. It is reported that 2–4% of car-kilometres can be switched to public transport by 2030, increasing to 5–8% by 2050^57^. The intended shift to public transportation and active travel could be hindered by an ageing population, socioeconomic disparities, poor and unsafe infrastructure, inadequate rural connectivity^58,59^, and negative perceptions regarding protection, autonomy, prestige, frequency, and reliability^60–63^, requiring targeted interventions to promote behavioural change.

The UK’s Zero–Emission Vehicle (ZEV) mandate^53^, one of the most ambitious global frameworks for transitioning to electric vehicles (EVs), sets targets requiring 80% of new cars and 70% of new vans to be zero-emission by 2030, rising to 100% by 2035. This initiative, coupled with the planned phase–out of non–zero emission HGVs by 2040 and the electrification of rail lines, reflects the UK’s commitment to decarbonising its transport sector. However, several significant challenges threaten the successful realisation of these targets. The transition to EVs still faces barriers such as high upfront costs relative to internal combustion engine (ICE) vehicles, particularly for lower–income households^64^, though the price gap has narrowed considerably with the growth of the used-EV market. Recent data indicate that purchase price premium of an EV in 2023 cost about 40% more than ICE equivalents, down from approximately 50% in 2020^65^. The availability of public chargepoints in the UK has also expanded rapidly, with more than 53,600 devices recorded in January 2024, representing a 45% increase compared with the beginning of 2023^65^. The Department for Transport has targeted the roll–out of 6000 ultra–rapid charge points by 2035, including EV infrastructure on–street and in public car parks^52^. The placement and use of on–street charging may also have implications for pedestrian accessibility and safety, and so must be carefully considered when selecting locations. On the other hand, concerns about battery sustainability, including the extraction and disposal of raw materials and the scarcity of critical minerals^66^, pose ecological and scalability challenges^67,68^. For HGVs, both battery-electric and hydrogen options are under active development. Recent evidence indicates that deployment is currently dominated by battery-electric HGVs. Across Europe, there are already over 15,000 BEV HGVs on the roads compared with fewer than 200 hydrogen-fuelled vehicles, with 7500 new BEV registrations in 2024 alone compared to around 100 hydrogen units^69^. While electrification of rail lines is planned, alternative solutions will be necessary where electrification is not feasible. The quieter operation of EVs may also raise safety concerns for pedestrians and other road users, particularly in urban areas^58^. Addressing these barriers will require comprehensive and integrated strategies that balance technological innovation, infrastructure development, and socioeconomic considerations to ensure the successful implementation of the ZEV mandate and the broader decarbonisation of the UK’s transport system.

The decarbonisation strategies outlined in the reviewed local and regional authority roadmaps share significant similarities. The regional transport roadmaps reflect diverse priorities and challenges tailored to the unique needs of their populations and economies while aligning with national decarbonisation goals. Predominantly rural areas such as Northern Ireland, Scotland, and Northeast England^70–72^ emphasise solutions for dispersed populations and car dependency, whereas medium- to high-density regions like London, the West Midlands, and the West of England prioritise mass transit and integrated public transport systems^73–75^. A common focus across all regions is the transition to sustainable transport modes, including walking, cycling, public transport, and innovative options like carpooling and on-demand services, with an emphasis on inclusive accessibility to reduce transport inequalities. Despite these efforts, shared challenges persist, including the need to decarbonise transport infrastructure, address the impact of heavier electric vehicles (EVs) on road durability, and ensure grid resilience and equitable access to EV charging for underserved populations^74,76^. Changing entrenched behaviours around private vehicle ownership remains a significant obstacle, with many regions adopting financial incentives, congestion charges, and public awareness campaigns to encourage alternatives^77^. Furthermore, long-term funding constraints and fragmented integration across modes and jurisdictions undermine the ability to deliver seamless and equitable mobility solutions. While some regions actively explore emerging technologies like hydrogen freight^72^, Mobility as a Service (MaaS), and autonomous systems^78,79^, others remain cautious, reflecting disparities in investment capacities and risk appetites. Addressing these challenges will require cohesive, locally informed strategies that balance the urgency of decarbonisation with regional contexts, ensuring transport systems across the UK evolve sustainably while meeting national climate goals.

Local authorities have also developed strategies to improve transport infrastructure, reduce carbon emissions, and achieve net-zero goals while enhancing citizen wellbeing, as outlined in Table 2. These strategies, however, face significant challenges due to varying carbon baselines across councils (e.g. 1990 for East Renfrewshire, 2005 for Leeds, and 2019 for Cornwall) and constraints on local authority budgets, which affect both the implementation of new transport strategies and the maintenance of existing infrastructure. Key gaps in the proposed strategies include an over-reliance on EVs, inequitable access to charging infrastructure, limited driving range, and concerns about battery longevity and replacement. Moreover, constraints on local budgets, often reliant on central funding, further complicate the ability of councils to implement ambitious transport strategies, highlighting the need for increased investment and innovative financing solutions to overcome these challenges. In the reviewed local authority roadmaps, renewable energy measures included both onsite generation (e.g. rooftop solar photovoltaic systems) and offsite procurement (e.g. power purchase agreements and green tariffs), indicating that local authorities are pursuing both approaches.

**Table 2:** Net zero strategies within local authorities’ road maps

Transportation technologies	Travel behaviour	Energy and materials
Electric vehicles	Shift to public transportation	Renewable energy
Autonomous vehicles	Active travel	Alternative fuels e.g. hydrogen
Shared micro-mobility services e.g. e-bikes and e-scooters	20-minute neighbourhoods	Low carbon construction materials e.g. warm asphalt mixes, recycled building materials.
Mobility as a Service (MaaS)	Reduction in car kilometres	
Smartphone apps e.g. smart ticketing	Car permits	
	Low emission zones	

**Fig. 5:**
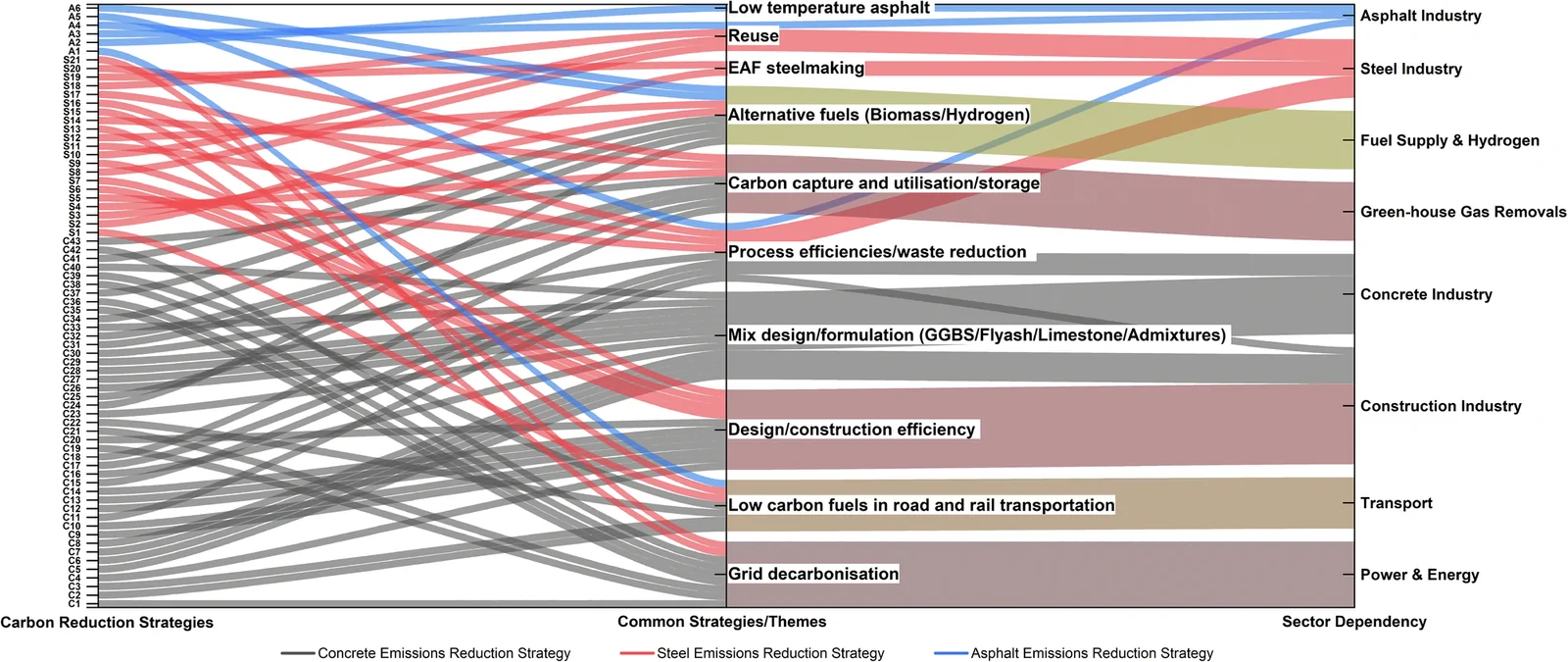
Mapping of carbon reduction strategies across the asphalt (A), steel (S), and concrete/cement (C) industries, showing sector-specific and shared approaches. See Table S2 for a detailed list of associated strategies.

Transport roadmaps have conventionally focused on reducing operational carbon emissions associated with traffic movement, often overlooking the embodied carbon linked to the construction and maintenance of physical infrastructure. Nevertheless, multiple roadmaps have been developed by transport authorities, industries, and institutions to decarbonise carbon-intensive materials such as concrete, steel, and asphalt^80–87^. Across these roadmaps, key strategies (listed in Table S2) are interconnected and dependent on various sectors and industries (Fig. 5). The power and energy sector plays a vital role in decarbonising construction materials, as the most widely adopted strategy—grid decarbonisation—relies on providing clean energy for energy-intensive processes in steel and cement manufacturing. Additionally, the availability of alternative fuels such as biomass or hydrogen is essential for reducing emissions in the production of construction materials (Fig. 5). Other commonly adopted strategies include reducing emissions from material transport within the transport sector and implementing carbon capture, utilisation, and storage (CCUS) technologies (Fig. 5). Technologies such as CCUS are yet to be realised with limited use to date, and raise many questions regarding the scalability along with their implementation cost. These interdependent strategies depend on external sectors beyond material manufacturing, and the material manufacturers play a significant role in influencing their supply chains and collaborating across industries to adopt these technologies.

Some strategies are common within the material and construction industries, such as process efficiencies, waste reduction, and design/construction efficiency, which can lead to substantial reductions in energy use and emissions. Such actions could lead to a 22% carbon reduction by 2050. For material-dependent strategies, the concrete and cement industry rely heavily on optimizing the mix design to produce low carbon concrete by replacing Portland cement with supplementary cementitious materials (SCMs) like ground granulated blast furnace slag (GGBS), fly ash, limestone fillers, and other admixtures. However, recent climate change initiatives have led to supply shortages for GGBS and fly ash, prompting the exploration of alternatives such as China clay (kaolin) from southwest UK quarries. While low carbon cements and concrete are the most widespread strategies, it could only reduce the 2018 baseline by 12%. In the steel industry, key strategies focus on circular economy principles and electric arc furnace (EAF) steelmaking. Meanwhile, for the asphalt industry, low-temperature asphalt production is a primary strategy for energy and carbon reduction. Therefore, in order for construction materials to achieve net zero, a tailored approach to decarbonisation is needed that considers both sector-specific challenges and broader sector dependencies.

### Digitalisation and technology

The National Infrastructure Commission (NIC) highlighted the strategic importance of leveraging technology and data to enhance infrastructure management, with the dual objectives of delivering consumer benefits and achieving cost efficiencies^88^. Key opportunities included the use of sensors and machine learning-driven analytics to optimise asset management and maintenance, minimise delays on transport networks, and utilise real-time data streams throughout the lifecycle of infrastructure assets to improve performance and safety. However, translating these ambitions into actionable strategies remains a challenge at the regional and local levels, where powers and budgets are often devolved. Similarly, the Geospatial Commission (GC) has underscored the transformative potential of geospatial data, identifying transport as a key focus area in its 2030 strategy^89^.

**Fig. 6:**
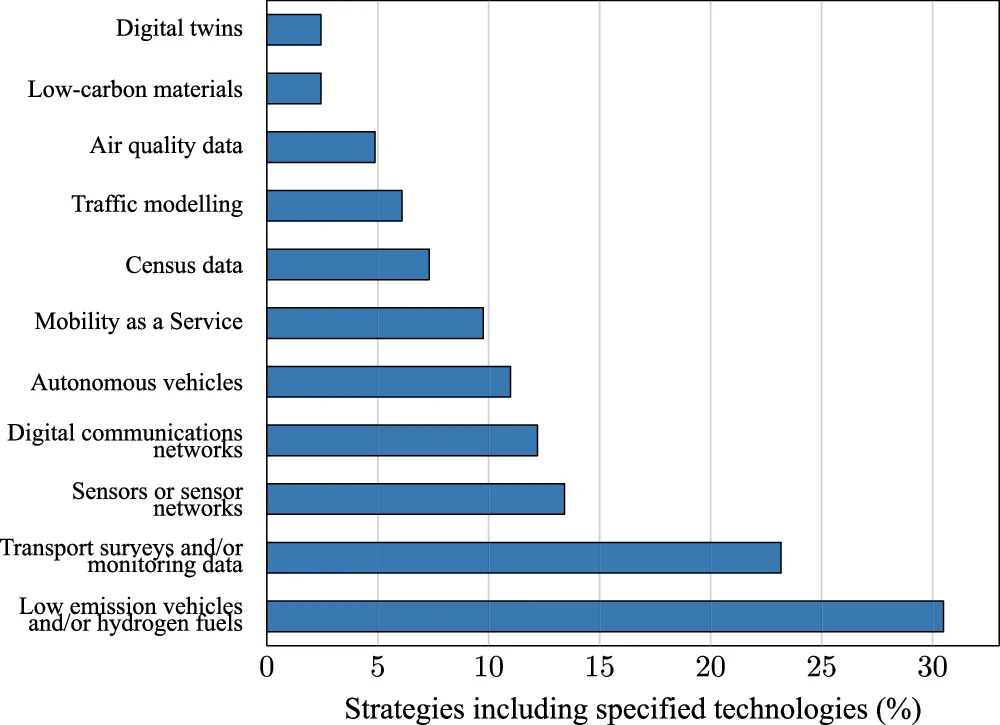
Specific technologies and data types identified in the strategy documents.

The role of technology and data in relation to meeting organisations’ strategic objectives was explicitly defined in only 27% of the reviewed documents across different levels (see Fig. 6), indicating a lack of specificity in articulating their strategic importance. Where technology and data were mentioned, they were often presented as general examples without a clear connection to organisational goals. 22% explicitly stated that organisations already had access to the data required to achieve their objectives, while 27% acknowledged a current lack of data but outlined strategies to acquire it in the future. The remaining documents indicated gaps in organisational data plans, potentially limiting their capacity to meet strategic goals. However, it is recognised that these gaps may be addressed in supplementary documents that were outside the scope of this review. These findings highlight the need for clearer integration of data strategies into local and regional transport plans to fully realise the potential of technological and geospatial innovations.

Among the reviewed documents that linked technology or data to specific objectives, the most common focus was the transition to net zero carbon emissions. This was discussed in 23% of the documents, which typically focused on the use of low-carbon vehicles or fuels such as electric or hydrogen-powered vehicles. Other goals included improving transport service quality through smart city applications, with 16% referencing technologies aimed at service users, such as contactless payments and smart ticketing, and 15% highlighting applications for planning and operational decision-making, such as tracking road demand or optimising traffic routing. Figure 6 shows the most common technologies identified in the review. While 30% of documents specifically referenced low-emission vehicles as critical to achieving net zero objectives, only 13% mentioned sensors or sensor networks, and just 5% identified specific applications of sensing technologies, such as infrastructure condition monitoring. These findings highlight significant gaps in the integration of technology and data into strategic planning, particularly in leveraging advanced tools to improve infrastructure performance and achieve long-term sustainability goals.

Few documents detailed the scale of current technology or data use, although some set future deployment targets, such as the planned rollout of EV charging infrastructure. This reflects the need for greater clarity in government policies on low- and zero-emission vehicles, alongside the expansion of the electricity system to support widespread EV adoption and the integration of circular strategies to secure critical materials for battery supply chains. By 2035, the UK aims to make EV batteries 95% recyclable and increase road vehicle occupancy through Transport as a Service (TaaS) models^52^. EV battery technology is rapidly evolving, with a shift from cobalt-heavy nickel manganese cobalt oxide (NMC) batteries to higher nickel content variants and lithium iron phosphate (LFP) chemistry for short-range vehicles, driven by cost and supply vulnerabilities^90,91^. Research into advanced battery technologies offers opportunities to surpass the theoretical limits of lithium-ion batteries^92^, yet significant gaps remain in the UK’s battery recycling and manufacturing industry. Developing a robust recycling framework and attracting gigafactories would strengthen supply chains and support net zero targets^93^.

A recent report to the UK Parliament on emissions reduction progress^94^ emphasised the importance of incorporating shared transport into planning reforms, particularly as advancements in autonomous vehicle technology support the shift toward TaaS and shared mobility models. Autonomous vehicles integrated into shared mobility schemes have the potential to enhance fleet management, improve vehicle efficiency, and reduce emissions^95–97^, but significant uncertainties remain regarding their impact on transport systems, road traffic patterns, private vehicle ownership, and infrastructure demands^98,99^. A modal shift away from private vehicles is critical for achieving the UK’s decarbonisation targets, with a reduction of 20–27% in private car use required by 2030^100^. Evidence from car-sharing schemes, such as car clubs, indicates their potential, with each car club vehicle replacing up to 20 private cars in 2021^101^. Case studies on carpooling, such as Lifeshare in the UK and BlaBlaCar in the EU, highlight their capacity to reduce private car use by increasing vehicle occupancy^102,103^. However, gaps in data persist, particularly for peer-to-peer car-sharing models, and there are concerns that shared mobility schemes may shift trips away from environmentally friendly modes like walking, cycling, and public transport, potentially undermining their environmental benefits^104^. These complexities underscore the need for further research to better understand the evolving role of shared mobility and its impact on decarbonisation, transport demand, and travel behaviour.

Nearly half (48%) of the reviewed strategies anticipated an increased rollout of technologies, with 30% assuming greater availability of lower-carbon fuels or vehicles in the future. Such assumptions are pivotal for achieving net–zero carbon targets, which frequently hinge on the adoption of lower–carbon technologies. However, any delays or shortcomings in these rollouts could substantially impede an organisation’s ability to fulfil its objectives. While many local and regional organisations may have limited influence over the pace or success of technology rollouts, the review revealed a concerning lack of acknowledgement of these risks and an almost complete absence of contingency planning. Notably, the Bristol Transport Strategy^105^ incorporated a contingency plan, which proposed prioritising investment in proven technologies to expand public transport infrastructure in the event that low-emission vehicles do not become sufficiently available. The absence of contingency plans in the majority of the reviewed strategies underscores a critical deficiency in risk management and adaptive planning across these documents.

Only 23% of reviewed documents referred to a clear data management strategy within their respective organisations, highlighting a potential area for improvement in how data are managed and utilised to support organisational objectives. Cybersecurity, a critical issue in increasingly digitalised transport infrastructure, was mentioned in just 10% of the documents, with limited detail provided. Notable strategies included the use of warm standby systems^50^ and references to the roles of government and industry in addressing cybersecurity threats and building capabilities^106^. This reflects a general underestimation of cybersecurity risks in transport operations and management. Furthermore, only 11% of the documents addressed the need for interoperable data systems, which are vital for the efficient operation and planning of integrated transport systems. Discussion on interoperability was often limited to identifying current barriers^106–108^, although one document outlined future plans for achieving interoperability in freight transport systems^109^. These findings suggest significant gaps in the integration of data strategies, cybersecurity measures, and interoperability planning, all of which are critical for advancing the digital transformation of transport infrastructure.

The review highlights significant data and technological gaps that constrain organisational operations and the effective delivery of transport services. Key data-related challenges include the rapid obsolescence of census data, limited insights into travel patterns, and insufficient information on the effectiveness of road safety initiatives. Technological priorities identified across the documents include the development of multi-operator, integrated, and equitable ticketing systems; next-generation signalling and predictive traffic management technologies; and tools to optimise kerbside space usage, particularly for deliveries. Other critical needs include predictive service information, such as forecasting the impacts of weather events on passengers, and greater clarity regarding the development and deployment of autonomous vehicles. These gaps underscore the necessity of enhanced data strategies and technological innovation to improve the efficiency, sustainability, and resilience of transport systems. However, the review also reveals a lack of clear definitions, robust planning, and contingency measures to address risks associated with future technology and interdependencies. Despite widespread acknowledgment of the importance of leveraging data and technology to achieve strategic goals, such as the transition to net zero carbon, many organisations appear underprepared to fully integrate these tools into their operations, potentially limiting their ability to achieve long-term objectives. Although artificial intelligence (AI) and machine learning (ML) were not explicitly referenced in the reviewed documents, their future role in transport technology, infrastructure management, and planning is likely to grow. The recently published UK Government Transport Artificial Intelligence Action Plan^110^ indicates a stronger policy emphasis on AI/ML adoption, which could encourage stakeholders to integrate these tools more explicitly into future transport strategies.

### Integrating updated climate science

Transportation is significantly influenced by climate change, with varied hazards such as precipitation extremes, severe thunderstorms, temperature changes, sea level rise, and wind patterns posing threats to infrastructure and operations. However, the integration of updated climate science into transport sector planning remains limited. Many sectoral documents fail to address specific climate hazards or rely on outdated climate models, creating a disconnect between advancements in climate science and their application to transport resilience and adaptation strategies. Only 5% of transport strategies use the latest UKCP18 climate projections, while most still rely on the older UKCP09 data or fail to specify the models used. This lack of specificity undermines the ability to assess and mitigate risks effectively, particularly as climate science has advanced significantly over the last decade.

Recent developments in high-resolution climate modelling, such as kilometre-scale convection-permitting models (CPMs), offer transformative insights for understanding localised climate impacts^111,112^. For example, the UKCP18-Local simulation suite provides 100 years' worth of simulations of the UK climate (from 1980 to 2080) on a 2.2 km grid, which allows convective rainfall events to be captured, and in turn projects an enormous increase in rainfall extremes with warming [e.g.^113^]. Earlier lower-resolution (~10–100 km grid) regional models with parameterised convection were producing less uplift in these extremes, and these new results warrant a requirement for a re-evaluation of flood risks for every sector, including transportation [e.g.^114^]. These advancements extend beyond precipitation, with CPMs providing better representations of hazardous weather events, such as thunderstorms, wind patterns, and upper-atmosphere turbulence, which are critical for both surface and aviation transport systems [e.g.^115–117^]. Moreover, emerging tools like machine learning and artificial intelligence are beginning to enhance climate projections, offering faster and computationally efficient ways to analyse risks, though their adoption in the transport sector remains minimal [e.g.^118^].

Despite these advancements, only a few transport authorities incorporate updated climate science into strategic planning. For example, hazards like heatwaves, which significantly affect road surfaces, rail infrastructure, and non-motorised transport, are under-represented in sectoral documents. Similarly, the role of compound hazards, such as simultaneous flooding and wind storms, is largely overlooked, with only 5% of documents addressing them. This lack of integration reflects broader gaps in contingency planning and risk assessment, leaving the sector ill-prepared to address the cascading impacts of climate-induced disruptions. Overall, 86% of the reviewed documents do not include specific information on the approaches and climate models used. While approximately two-thirds of the documents reviewed mention climate change, the impacts of climate change are often only briefly mentioned with generic terms like ‘climate risks’, ‘climate crisis,’ etc. Among those hazards specified, flooding accounts for the largest share, as shown earlier in Fig. 1. Other hazards include heatwaves, droughts, fires, turbulence, icing (for aviation), sea level rise, sea fog, sea currents, ice hazards (over oceans), storms, and wintertime wind/rain, although these latter hazards are much less commonly addressed within the strategy documents.

The transport sector must integrate the latest advancements in climate science into its planning processes, leveraging tools such as UKCP18^25^, convection-permitting models (CPMs)^112^, and machine learning^119^ to enhance vulnerability assessments and resilience strategies. This alignment should prioritise a comprehensive evaluation of transport systems’ exposure to both current and future hazards, using improved insights into climate extremes to guide adaptive and proactive measures. Collaboration between the transport sector and the climate science community is essential to bridge knowledge gaps, foster innovation, and ensure that planning remains data-driven and future-focused. Without such integration, the sector risks being ill-prepared to tackle the complex and interconnected challenges posed by climate change, undermining efforts to develop sustainable and resilient transport systems.

### Views from stakeholders

The insights from the review reveal critical gaps and opportunities in resilience planning, decarbonisation, digitalisation, and climate science integration. These findings directly informed the workshop discussions, which explored how such challenges are perceived and prioritised by stakeholders. The workshops, which engaged over 100 stakeholders, including policymakers, practitioners, and other key actors, revealed that the challenges of achieving resilience and decarbonisation within the UK transport infrastructure sector vary significantly across national, regional, and local contexts. The following synthesis reflects the themes most consistently raised across participants, while recognising that some views diverged. Each level presents distinct priorities, complexities, and opportunities for intervention, highlighting the need for targeted strategies that address the specific needs and characteristics of each scale.

At the national level, participants emphasised that the primary challenges focus on overarching issues such as energy dependence, governance, and system interdependence. Transitioning to low-carbon energy sources remains a critical priority to align the transport system with net-zero targets. System interdependencies, such as the cascading effects of disruptions across energy, transport, and communication networks, require coordinated national policies and investments in infrastructure resilience. Governance challenges are particularly acute at this scale, as they involve balancing conflicting priorities across sectors and aligning national transport policies with international climate commitments. The development of a unified funding strategy is essential, addressing uncertainties around long-term financial support for infrastructure projects that deliver both resilience and decarbonisation. Additionally, tackling systemic issues, such as ageing national transport assets, requires a consistent approach to modernisation and integration with climate resilience objectives.

At the regional level, workshop discussions highlighted challenges centred around addressing infrastructure interdependencies, regional climate hazards, and governance boundaries. Regions must navigate complex interactions between transport networks and other systems, such as energy grids and water infrastructure, particularly when planning for cascading impacts of extreme weather events^120^. Regional transport strategies also face the challenge of addressing spatial inequalities, such as the lack of adequate rural transport options and the uneven distribution of infrastructure investments. Governance coordination is a particularly significant challenge at this scale, where regional boundaries often complicate the alignment of policies and funding mechanisms. For example, coordinating between sub-national transport bodies and local authorities can help ensure that investment and planning priorities are consistent across regions. Addressing regional climate risks, such as flooding and extreme heat, is another critical area, requiring the integration of regional climate adaptation measures into transport resilience strategies.

Locally, participants noted that challenges focus on community engagement, immediate transport needs, and practical implementation barriers. At this level, issues such as public perception, behavioural change, and making the case for funding availability and clarity are particularly pressing. Local authorities often face resource constraints, yet they are responsible for addressing visible and immediate challenges such as poor road maintenance, a lack of active travel options, and community buy-in for transport initiatives. Demand modelling and the coordination of maintenance and capital works are often easier to implement at the local level but require strong local leadership and clear alignment with regional and national priorities. Additionally, local adaptation to demographic and socioeconomic changes, such as shifting population needs and mobility demands, highlights the importance of flexible and responsive transport planning. Local contexts also provide opportunities for innovation, such as piloting low-carbon technologies and embedding monitoring systems to enhance resilience. However, these initiatives often face barriers such as insufficient technical expertise, funding uncertainties, and difficulties in scaling successful pilots to regional or national levels.

Critical gaps and barriers to the effective use of data and technology in the transport sector were also raised by participants across workshops. Foremost is the need for standardised transport data that can be utilised across the sector, adhering to the FAIR principles^121^, with a presumption in favour of open-access data. This must be complemented by open-source tools and models that can operate at different levels to facilitate the interpretation and application of these data. Additionally, harmonised approaches to data collection and reporting are required to ensure consistency across the sector, alongside efforts to overcome barriers to data sharing and improve data interoperability. Standards within the sector must also evolve to accommodate emerging and future technologies. The workshops further highlighted the importance of identifying reliable data sources that describe the resilience of infrastructure, which could inform resilience standards for the sector. A significant gap exists in the availability of real-time, network-level data to optimise routing and re-routing during disruptions. Similarly, there is a lack of real-time data on asset condition, loading, capacity, and deterioration, which could be addressed through structural health monitoring (SHM) systems and sensor networks. Finally, the absence of real-time data on construction and maintenance operations, such as crew availability and equipment condition, presents an additional barrier to effective decision-making and operational efficiency in the transport sector. Addressing these gaps is essential for enhancing the sector’s data-driven capabilities and resilience.

Across all scales, a consistent theme is the need for improved integration and collaboration. Workshop participants stressed that national policies must provide clear guidance and resources that empower regional and local authorities to implement effective solutions. At the same time, local innovations and insights should inform regional and national strategies to ensure policies are grounded in practical realities. Challenges such as public perception, behavioural change, and governance inefficiencies require multi-level coordination to ensure that transport systems evolve cohesively and sustainably. The findings further underscore the need for collaborative approaches to overcome barriers. For example, resolving conflicts between local, regional, and national transport ambitions will require improved governance structures, shared funding models, and active public and private sector engagement. The integration of advanced technologies, such as embedded monitoring systems and digitisation for better assessment, offers pathways for enhancing system resilience, but these need to be aligned with standards and practices across different sectors.

The stakeholder perspectives highlight how the challenges and opportunities identified in the review are experienced differently at national, regional, and local levels. These insights point to the need for a decision-making framework that links evidence to action. Some challenges, such as demand modelling and coordination of maintenance works, can be addressed relatively quickly, whereas others, including governance reform, funding clarity, and public perception, require sustained efforts. Strategic interventions must therefore reconcile short-term priorities with longer-term systemic change, ensuring that the transport system evolves in alignment with net-zero goals and can withstand future shocks. This calls for an iterative and collaborative approach that incorporates stakeholder insights and embeds decarbonisation and resilience across all aspects of transport planning and operations.

### Decision-making for decarbonised, adaptable and resilient transport systems

**Fig. 7:**
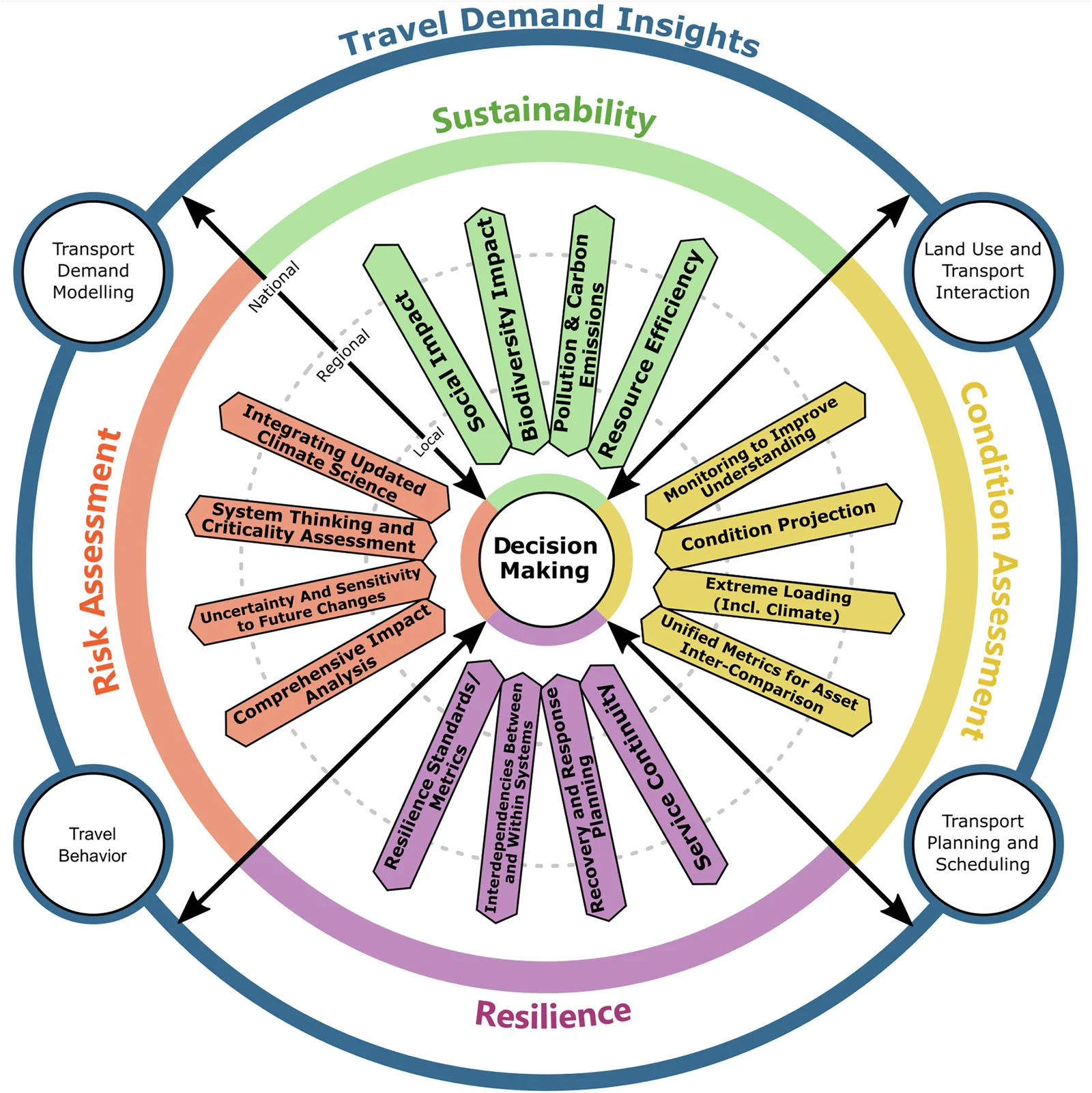
Conceptual approach to holistic decision-making across different governance levels. Each of the ‘fingers’ radiating into the centre represents a specific analytical or planning activity that informs decision-making. For example, under Risk Assessment, these include integration of updated climate science, systems thinking and criticality assessment, uncertainty and sensitivity analysis, etc.

The findings from the review and stakeholder interactions underscore the urgent need for systematic decision-making procedures that integrate resilience and decarbonisation into the transport sector. This approach must consider diverse infrastructure asset types, system interdependencies, and varying hazards within transport planning, design, and operations. Furthermore, it must explicitly account for how the ability of transport infrastructure systems to deliver sufficient capacity directly influences travel demand, traveller behaviour, and responses to disruptions. In response to the gaps identified in the review, such as limited use of updated climate science, weak resilience standards, and insufficient attention to equity, and the challenges raised in the workshops, including governance barriers and behavioural change, these insights have been synthesised into a conceptual decision-making framework. This framework (Fig. 7) provides a practical guide for applying a holistic approach across different governance levels and is outlined below.

The proposed framework brings together the four segments of risk assessment, sustainability, condition assessment, and resilience, each associated with the capacity of the infrastructure system. Each segment is operationalised through a set of analytical components, or ‘fingers’, while travel demand insights act as the integrative loop that connects them. The loop captures both how users respond to policies or disruptions and how broader patterns of travel behaviour, land use, and demographic or technological change shape infrastructure needs over time. The framework is intended as a practical guide to support decision-making rather than a purely theoretical construct. When applying it, the decision-makers first establish the context (for instance, a local transport investment plan, a regional transport strategy, or a national decarbonisation roadmap) before working systematically through the components of each segment. The ‘fingers’ serve as a checklist of analytical tasks, ensuring that critical issues are not overlooked and the process is designed to be iterative, offering a structured way to prioritise interventions, compare alternatives, and revisit assumptions as conditions change. Table S3 lists the analytical components within each segment and describes their role in decision-making.

- **Risk assessment** should evaluate infrastructure risks not only in terms of asset performance but also by considering multi-dimensional impacts, including safety, environmental, social, and economic consequences^122,123^. Such assessments must account for uncertainties across various future scenarios (e.g. shifts in loading conditions, variations in extreme weather events, and long-term climate change)^124^. Adopting a whole-system perspective of transport is imperative, incorporating criticality assessments across multiple spatial scales (from individual assets to network-wide analysis)^119,125^ and across temporal horizons, ranging from immediate emergency responses^126^ to long-term strategic planning^127,128^. Identifying critical nodes^129,130^ and vulnerable points^131^ enables stakeholders to evaluate both localised and systemic risks, facilitating effective management and contingency planning. The risk assessment segment within the framework also emphasises the need to integrate updated climate science, apply systems thinking, and use sensitivity analysis to test the robustness of candidate strategies under a wide range of uncertain futures.
- **Sustainability**: Treating sustainability as an afterthought often leads to cost overruns and implementation failures^132^. Sustainability extends beyond carbon emissions to encompass factors such as resource efficiency^133^, circularity^134^, biodiversity^135^, and the reduction of other pollutants such as microplastics, air and noise pollution^136^. It also includes a social dimension, ensuring that issues of equity, inclusivity, affordability, and accessibility are addressed alongside environmental and economic considerations. To facilitate this, these factors should be represented as measurable metrics, enabling easier incorporation into planning and informed decision-making. In new construction projects, embedding sustainability is more straightforward, as targets and behavioural expectations can be established early in the project lifecycle^137^. However, on the operational side, incorporating sustainability considerations requires greater support and well-defined guidelines^138^. Budget constraints, supply chain availability, and uncertainties against performance of novel low carbon materials and technologies add complexity, underscoring the importance of clear and actionable strategies. Early integration of sustainability into projects has consistently proven beneficial^132^. In addition to embodied carbon associated with infrastructure, there is also a need to incorporate future mobility scenarios such as uptake of EVs^139^.
- **Resilience and adaptation**: Traditional transportation planning has predominantly prioritised operational efficiency, often neglecting essential aspects of resilience and the complex interdependencies within critical infrastructure systems^130,140^. In response to the increasing frequency and severity of weather-related events, there is a growing recognition of the need to explicitly integrate resilience and adaptation measures into transportation planning and design, enabling systems to better anticipate disruptions, withstand disturbances, and rapidly recover to ensure continuous service provision and safeguard economic stability^141^. Adaptation strategies, informed by up-to-date climate science^142^, can guide infrastructure modifications, investment priorities, and operational improvements to better manage risks from extreme weather events, long-term climatic shifts, and other disruptive events. Prioritizing investments that build redundancy, such as alternative routes or multi-modal transport solutions^143^, can also strengthen system robustness and continuity during disruptions. Effective resilience planning requires systemic thinking, assessing vulnerabilities not only at the asset level but also across networks^144^, considering interdependencies between transportation infrastructure and other critical systems such as energy and telecommunications^130^. Within the framework, resilience could be further operationalised through the establishment of resilience standards, explicit recovery and response planning, and the incorporation of service continuity measures that ensure transport systems remain functional during shocks. Such proactive approaches not only safeguard infrastructure assets but also promote equitable outcomes by reducing vulnerability and ensuring reliable transport accessibility for all communities.
- **Condition assessment**: To effectively manage transport infrastructure systems and their associated assets, it is essential to understand their current condition and how this condition may evolve over time [see, for example,^145–147^]. This task is inherently complex due to diverse asset types that may require unique condition measures and are subject to varying modes and mechanisms of deterioration. Additional complexities arise from the variable loading that transport systems experience over time, influenced by factors such as climate change (which alters the frequency and magnitude of hazards), other natural and anthropogenic risks, and shifts in demographic, economic, and technological landscapes. To this end, transport infrastructure management must incorporate robust asset condition assessments, along with reliable forecasts and projections of future condition changes [see, for example,^46,148^] that account for projected future loadings and the likely capacity requirements. They must also produce outputs that enable comparisons across multiple asset types, often by translating condition data into metrics such as risks of failure. These failures may be defined in terms of ultimate or serviceability limit states, depending on the specific requirements of the transport system and the legislative regime in which it operates. The framework highlights the importance of enhancing condition assessment through improved monitoring, forecasting, and the use of unified metrics that allow for prioritisation of assets and networks.

Understanding and modelling travel demand is critical to developing resilient and low–carbon transport systems since it directly influences infrastructure usage, stress and the evolution of travel behaviours, and therefore contributes to understanding capacity requirements that the transport infrastructure needs to support. Traditional methodologies, such as the four–step approach and four–step models (FSMs), have primarily focussed on aggregated motorised travel behaviours at regional or sub–regional scales^149–151^. However, these conventional models are increasingly seen as inadequate, as they restrict the range of trip activities and often fail to capture the complexity of contemporary mobility patterns—particularly the emergence of alternative modes such as e-bikes, shared mobility services and active travel^152–155^. Furthermore, traditional models generally overlook the intricate spatial and temporal interdependencies inherent in individual travel behaviours and household dynamics^156,157^. In response, recent advances in data analytics and computational techniques have led to the development of agent-based models (AgBMs), which simulate individual travel decisions by integrating detailed socio-demographic attributes and comprehensive daily activity patterns in space and time^155,158,159^. These models tend to focus on three primary scenarios:

- Analyse general transport mobility, including congestion management, logistics, and travel behaviours under typical conditions^160–162^.
- Evaluate the effectiveness of specific policy interventions such as promoting public transport^162–165^, adopting sustainable mobility practices^166,167^, expanding the market share of electric vehicles^168–170^, and integrating automated mobility on demand^171,172^ before their real–world implementation.
- Examine how extreme weather events may affect both transport networks and the wider population, thereby highlighting the need for robust resilience planning^173–177^.

While the first scenario concentrates on capturing routine mobility patterns encompassing different user groups, transport modes, and travel schedules, the other two scenarios explore ‘what if’ conditions in which decarbonisation policies or hazardous climate events transform existing travel practices and require individuals to adapt to new realities. This bottom-up approach provides a more nuanced and realistic representation of travel demand, thereby offering critical insights for the formulation of sustainable mobility policies and response of transport users to disruptions.

Additionally, capturing how travel behaviours change during and after disruptions is crucial, as it directly impacts the capacity requirements of the wider transport system and individuals’ ability to move effectively. Understanding these behavioural shifts can inform infrastructure planning and operational strategies to ensure that transport networks remain functional and resilient under stress conditions. During disruptions, transport users may adopt alternative modes or routes, adjust their travel timing, or choose not to travel altogether^178^. These adjustments not only redistribute demand across different parts of the network but also influence future travel choices and patterns. By accurately modelling these dynamic adjustments in travel behaviour, transport planners and policymakers can better predict network performance and identify necessary investments or interventions to maintain system functionality, accessibility, and equity in mobility during and after disruptive events^179^.

## Discussion

The UK’s transport sector is dealing with multiple challenges, such as the pressing need to decarbonise and build resilience, integrate advanced technologies and data, and align with the updated climate science. The resilience of the UK’s future transport systems depends on comprehensive measures to mitigate the impacts of extreme weather and enhance infrastructure durability. The Department for Transport’s Transport Resilience Review^180^ provides recommendations for strengthening transport systems against adverse weather events, emphasising the need for robust integration, proactive maintenance, and strategic investment. Work is ongoing to address issues related to the adaptation of the UK’s transport infrastructure to climate change, as discussed in the DfT consultation document ‘Fit for a changing climate? Adapting the UK’s transport system’^181^, the responses to which are currently being reviewed by the UK Government. The challenges posed by ageing transport infrastructure are often amplified by the significant age of many existing assets. For example, Network Rail, owner and manager of Great Britain’s mainline rail network, estimates that a substantial proportion of the UK’s railway earthwork assets are over 150 years old, with some exceeding 170 years^44^. These assets were constructed long before the advent of modern construction equipment, with minimal documentation of construction specifics and predating the development of modern soil mechanics. Combined with weather-driven cyclic deterioration^182,183^, this creates significant uncertainty regarding their current condition and how they may respond to future loadings, and this issue will become more pressing with time as existing assets continue to age, deteriorate and require progressively increasing levels of maintenance and renewal.

The UK Government’s Resilience Framework^184^ proposes a strategic, society-wide approach, highlighting resilience standards and cross-sector collaboration. Specific actions include a £5.2 billion investment in flood and coastal defences and integrating climate resilience into industrial and security strategies. Moreover, the government’s National Adaptation Programme^185^ underscores the necessity for proactive adaptation across all areas of transport policy, supported by a £15 million joint research initiative aimed at equipping researchers and policymakers with essential data and skills. An additional and often under-recognised challenge relates to workforce availability and skills capacity within the transport sector. Delivering and maintaining resilient, decarbonised, and digitally enabled transport systems requires a wide range of expertise, including electricians, civil and electrical engineers, project managers, IT specialists, and data scientists. Current evidence suggests that the UK, similar to many other developed economies, is experiencing shortages in several of these critical skill areas^21,186^. Such gaps risk delaying infrastructure projects, constraining the roll-out of new technologies, and reducing the overall resilience of transport systems by limiting their capacity to respond to shocks and undertake timely maintenance. Addressing these skills gaps will require coordinated strategies across government, industry, and academia, including investment in training, apprenticeships, and retention initiatives to ensure a sufficiently skilled workforce for both current and future demands.

Currently, there is no statutory requirement in the UK mandating specific resilience targets, although there is an aspiration to establish and implement these by 2027^181^. The absence of formal resilience targets, especially when compared to existing quantitative statutory requirements for reducing carbon emissions, can partly be explained by the relative urgency attached to mitigating greenhouse gas emissions compared with addressing resilience challenges in infrastructure systems. Additionally, defining uniform resilience standards is particularly challenging due to the inherent diversity and complexity of transport infrastructure assets and systems, each having distinct vulnerabilities, operational characteristics, and risk profiles, as discussed by the NIC^186^. Further complicating this situation is the issue that, where statutory resilience requirements are defined, they tend to be purely qualitative/descriptive [see, for example, the licence for UK National Highways;^187,188^]. As such, the current lack of universally accepted resilience performance indicators limits the ability to make meaningful comparisons between different assets within, and across, transport systems. This gap poses a considerable obstacle to effective integrated asset management, particularly given the interdependencies across sectors such as transport and energy, which must also be considered in resilience planning.

The Department for Transport’s Future of Transport programme^189^ emphasises the development and deployment of zero-emission vehicles (ZEVs), autonomous and connected vehicles, and the integration of digital technologies to enhance travel efficiency and safety. Central to this initiative is the Transport Decarbonisation Plan^52^, outlining ambitious targets to achieve net-zero emissions by 2050 through expanding electric vehicle charging infrastructure, promoting active travel modes, and advancing innovative digital solutions. Accordingly, the UK government’s urban mobility strategy^18^ prioritises innovation, encouraging active travel, reducing congestion, and supporting a market for new transport services, such as the ArrivaClick on-demand service^155^. The shift towards ZEVs is projected to cut CO_2_ emissions by 80% by 2050 and reduce NOx and PM10 emissions by 95% and 98%, respectively, delivering major air quality improvements and public health benefits^52^^,53^. For every £1 invested in urban mobility innovation, the estimated return is projected to be £8–£14 by 2025 and £20–£34 by 2030^190^. In contrast, rural areas, home to nearly 10 million people in the UK^191^, present distinct mobility challenges. The reliance on private vehicles restricts access to essential services, exacerbates social isolation, and negatively impacts mental health, particularly affecting the elderly, individuals with mobility constraints, and younger populations. Recognising these unique rural mobility issues, recent governmental initiatives have focused on alternative transport solutions, including e-bikes, demand-responsive transport, connected autonomous vehicles, and drone deliveries^19^. Successful examples include the HertsLynx demand-responsive service in Hertfordshire, e-cycle pilots in Cornwall, mobility-as-a-service applications in Scotland’s Highlands and Islands, and infrastructure restoration projects like Tadcaster Bridge. Initiatives such as the Rural Mobility Fund (£20 million) for trialling on-demand bus services^192^, Restoring Your Railway fund (£500 million) for reopening closed rail lines and stations^193^, expanding EV charging infrastructure through the LEVI fund (£450 million)^194^, and Zero Emission Bus funding (£129 million)^195^ reflect the UK’s commitment to addressing rural connectivity through targeted innovation, infrastructure improvements, and technology integration.

Current policies and standards governing material usage in the construction of transport infrastructure are stringent; however, several critical issues persist, suggesting either an insufficient awareness or lack of appropriate action to address them effectively. Despite rigorous regulations, important aspects such as carbon emissions associated with repair materials and infrastructure maintenance are frequently overlooked, undermining decarbonisation efforts. While advanced construction technologies and innovative techniques offer significant opportunities for optimising resource efficiency, their implementation is limited by inadequate investments in technology, workforce training, and constrained supply chain capacities. Transparent standards, certifications, and clear regulatory frameworks are critical in verifying their environmental benefits and quality, thereby enhancing market trust. Additionally, incentivising early adopters through financial support and favourable procurement practices will stimulate demand, encourage industry investment and catalyse scaling of production capacity. Collaboration among industry stakeholders, academia, policymakers and regulators fosters knowledge exchange and facilitates coordinated action, further reinforcing the supply chain. Finally, dedicated investment in research, innovation and skills development, coupled with proactive risk management strategies, will ensure sustained growth and resilience within the low-carbon materials sector.

Recent advancements in computational mechanics, artificial intelligence, data science, and sensor technologies have accelerated transport innovation, offering cleaner transportation options^196^, enhanced asset condition monitoring^197^, and improved capabilities to predict infrastructure performance under variable loading and deterioration conditions^148,198,199^. These developments facilitate a more accurate assessment of capacity requirements and resilience planning, particularly crucial given that urban areas are likely to experience the earliest and most pronounced changes in travel patterns due to their high economic activity and limited space^200^. The recent publication of the UK Government’s Transport Artificial Intelligence Action Plan^110^ further underscores the growing expectation that AI and machine learning will become integral to transport planning, infrastructure management, and service delivery. New and more advanced models are needed to account for the evolving mobility requirements, as there is a need to understand the impact of transport policies at a more granular level^201^, especially for a decarbonised and resilient transport sector. New approaches to mobility (e.g. MaaS^202^, Demand Responsive Transit, DRT^203^) and a greater diversity and heterogeneity of mobility patterns (e.g. depending on different sociodemographic attributes, attitudes, and trip purposes) require a more detailed analysis at the individual level. AgBMs are an alternative to traditional models to accomplish these mobility components. Although these models are developed and used extensively in research, their importance is currently gaining momentum in industry and national institutions. For instance, New Zealand^204^, Switzerland^205^ and Germany^206^ are currently using AgBMs in their transport demand modelling. Recently, the UK DfT included the use of AgBMs in the Transport Analysis Guidance (TAG) unit M5-4, as a consequence of the evolving requirements in transport modelling for strategic planning, exploring policy options and gathering evidence for strategic cases at the very early stage of business cases^201^. Although AgBMs are not without limitations such as data preprocessing, computational efficiency, and reproducibility challenges^158^^,207^^,208^, they offer a novel perspective in transport modelling that supports both decarbonisation targets and enhanced system resilience.

This study finds that current UK transport strategies demonstrate a strong commitment to decarbonisation, but a comparatively less developed approach to resilience and climate adaptation. Ambitious measures such as the widespread adoption of ZEVs, expansion of charging infrastructure, and promotion of active travel are projected to drastically cut transport emissions and yield co-benefits for air quality and economic growth. However, equivalent progress on resilience is lacking. There are still no binding resilience targets or widely adopted metrics to gauge how well transport systems can withstand climate extremes. This imbalance echoes the findings of other assessments such as the^209^ who recently observed that adaptation planning remains ‘piecemeal and disjointed’. This study reinforces this critique by showing that while decarbonisation plans are detailed and quantitative (e.g. clear timelines for EV rollout and carbon reduction), resilience efforts often rely on qualitative guidelines and reactive measures rather than measurable performance indicators.

Overall, this study contributes to research by providing one of the first integrated syntheses of UK transport strategies across governance levels, explicitly linking resilience, decarbonisation, digitalisation, and climate science. The proposed conceptual framework developed operationalises these insights into a practical guide for decision-making. For policy and practice, the framework provides a checklist of analytical components that can be applied at national, regional, and local scales, supporting alignment between strategic ambitions and on-the-ground implementation. For society, the emphasis on equity, rural accessibility, and behavioural change ensures that the transition to a net-zero transport system is inclusive and resilient.

While this study provides a comprehensive evidence base, several limitations must be acknowledged. The document review was limited to publicly available strategies and may not capture internal or unpublished processes. Transport policy evolves rapidly, meaning that some reviewed documents may become partially outdated as new strategies are issued. Nevertheless, the combination of review and stakeholder perspectives adds robustness by triangulating evidence across multiple sources. Moreover, the integration of national, regional, and local insights provides a rare multi-scalar perspective that strengthens the generalisability of the findings.

## Conclusions

The results of this study highlight the urgent need for a more integrated and systemic approach to transport planning than is currently practised. While net-zero strategies have received strong policy attention, they often overshadow equally important issues of resilience and adaptability. Current practice tends to treat the transport system in silos, with limited recognition of interdependencies within and between systems and insufficient consideration of their wider impacts on people and the environment. The analysis also reveals both persistent challenges and emerging opportunities. Key challenges include the limited use of updated climate science, weak or absent resilience standards, fragmented governance structures, and insufficient attention to equity in decision-making. Stakeholders further highlighted barriers related to behavioural change and the misalignment of priorities across governance levels. Opportunities, however, are also clear. Expanding active travel and investing in low-carbon public transport provide immediate routes to reduce emissions and enhance accessibility. Advances in digitalisation and data analytics can improve asset monitoring and predictive maintenance. Emerging technologies such as zero-emission vehicles and alternative fuels open further pathways, though they require careful planning to address affordability, energy capacity, and material supply risks. Strengthened collaboration between national, regional, and local authorities, supported by cross-sectoral partnerships, presents perhaps the most important opportunity: aligning decarbonisation and resilience objectives and translating strategic ambitions into coordinated action.

By addressing these challenges and capitalising on the identified opportunities, the proposed framework (Fig. 7) has the potential to guide both policymakers and practitioners towards a transport system that is not only decarbonised but also resilient, adaptable, and socially equitable, ensuring long-term benefits for society and the environment. Several areas for future research and improvements in processes and practice have been identified:

- **Integrated decision-making tools**: Practical tools should be developed to enable practitioners to apply systems thinking without being hindered by complexity, facilitating more effective integration of resilience, decarbonisation, equity, and adaptability in transport planning. These tools should underpin strategies and investment appraisal processes that better acknowledge social value, carbon reduction, and resilience benefits.
- **Robust data-sharing protocols and integrated performance measurement**: Establishing clear protocols and platforms for data sharing can enhance cooperation among sectors and stakeholders, supporting informed strategies. Investment in high-quality, transparent, and auditable data collection, particularly on carbon emissions, asset conditions, and predictive analytics, is vital. Comprehensive performance measurement frameworks that simultaneously address carbon reduction, resilience, equity, and adaptability are also required to guide continuous improvement.
- **Adaptive governance and capacity building**: Governance structures should facilitate integrated and devolved decision-making, encouraging flexibility and adaptability in standards to foster innovation. Investing in skill development is crucial to equip professionals with the capabilities needed for modern, integrated transport planning and implementation.
- **Public awareness and equitable provision**: Enhancing public awareness can drive behavioural change and promote sustainability objectives, ensuring a just transition. Transport provision must prioritise equity through inclusive planning processes and equitable investment strategies to ensure accessibility and social inclusion across all societal groups.
- **Energy considerations and freight integration**: Strategic transport planning must explicitly address energy provision constraints, particularly given increasing reliance on electrification, exploring alternative energy solutions. Additionally, freight transport should be integrated holistically into strategic planning efforts, given its substantial influence on the overall transport system.
- **Supply chain capability development**: Investment in strategies, business cases, and decision-making frameworks is necessary to build capacity and confidence in materials and technologies required for resilient, low-carbon transport provision.
- **Upskilling workforce**: Addressing workforce availability and skills gaps across engineering, digital, and project management roles is essential, as these directly affect the sector’s ability to deliver and maintain a decarbonised and resilient transport system.

The recommendations from this study advocate for a comprehensive shift towards integrated, systemic thinking in transport planning and the management of transport infrastructure networks at multiple scales, committing to a holistic system-of-systems approach. By addressing collaboration, data sharing, performance measurement, regulatory environments, public behaviour, equity, standards, predictive capabilities, energy constraints, and the inclusion of freight transport, these strategies aim to enhance the resilience, decarbonisation, and adaptability of transport systems. Implementing these recommendations can help ensure that transport infrastructure meets the complex challenges of the future in a sustainable and equitable manner.

## Methods

**Fig. 8:**
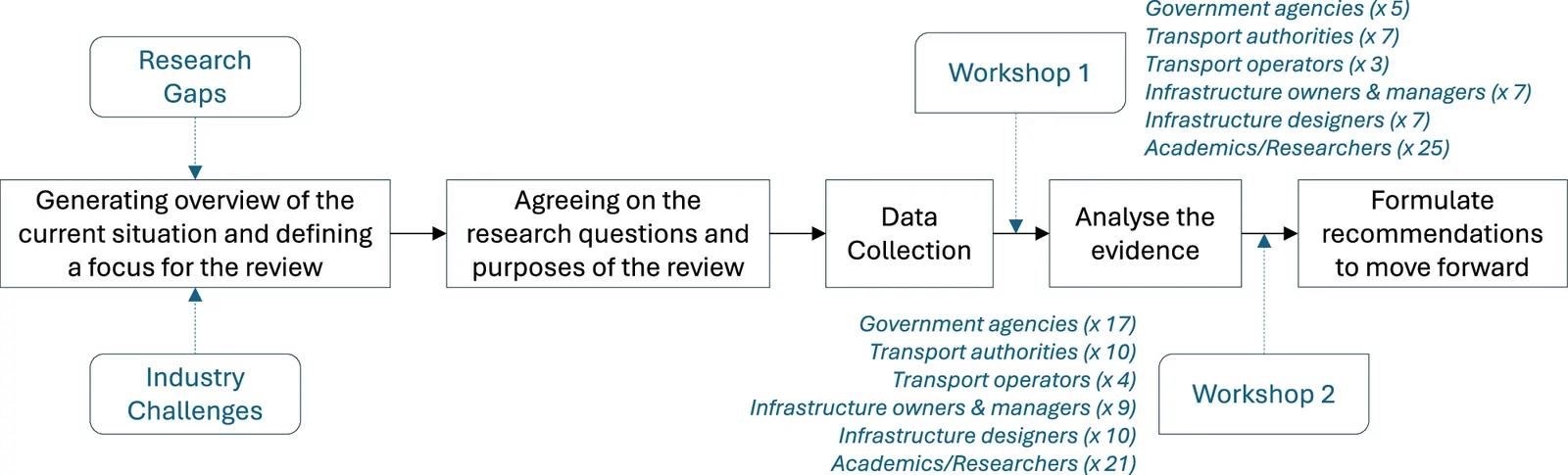
Flow chart of the research process.

This study is based on (i) a desk–based review of publicly available policies, strategy documents, and roadmaps issued at national, regional, and local levels in the UK, each addressing the decarbonisation and resilience of the transport system and (ii) a series of workshops including representatives from academia, national and local government, along with transport infrastructure owners, managers, and consultants where their views could be elicited and discussed. The research flow is presented in Fig. 8.

### Review of policy and strategy documents

**Fig. 9:**
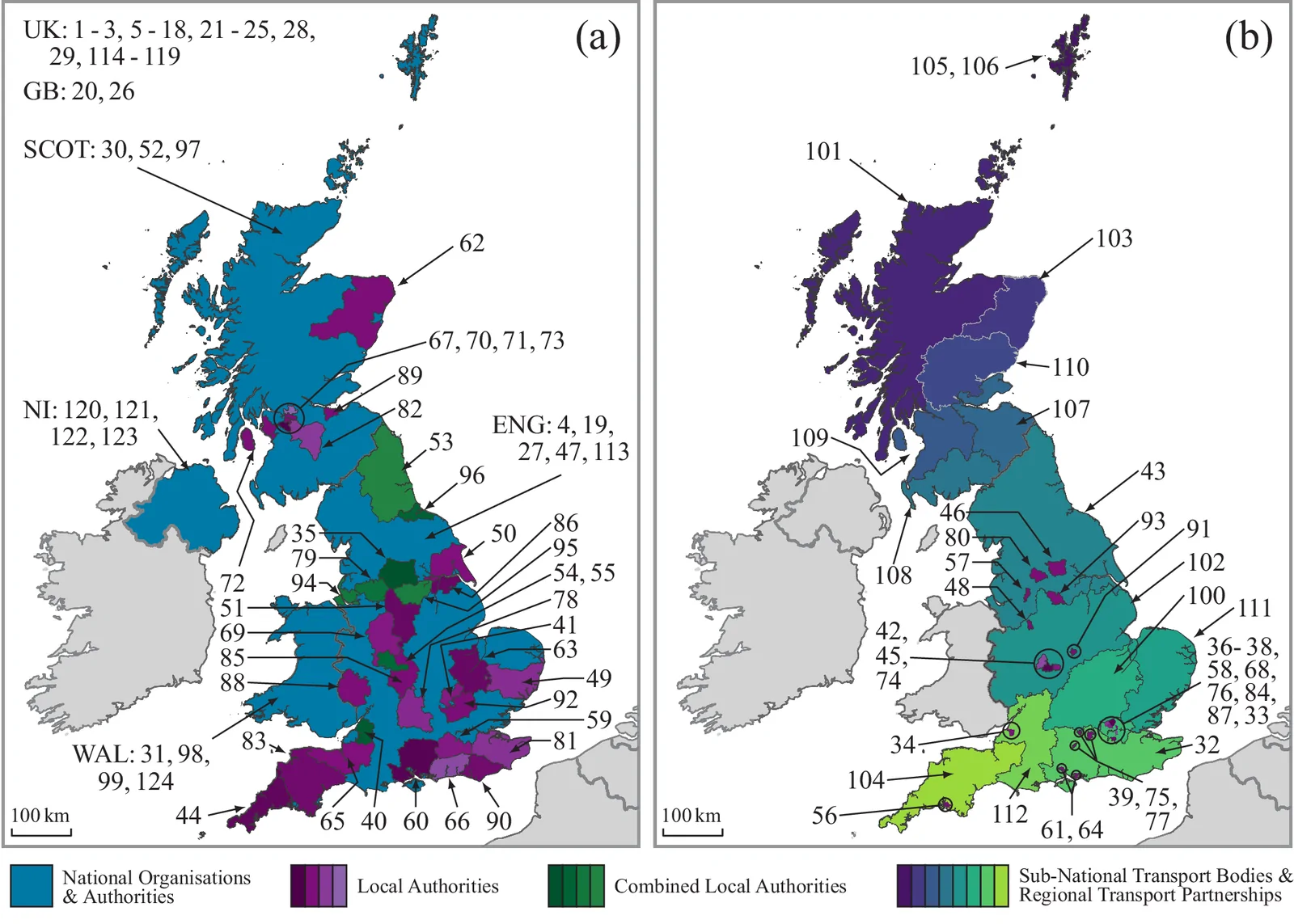
Geographic distribution of policy documents reviewed. The geographical distribution of the documents reviewed **a** national bodies, combined authorities, and large local authorities, and **b** sub-national transport bodies, regional transport partnerships, and smaller local authorities. Callouts indicate the relevant document(s) listed in the Supplementary Material. GIS data is sourced from the Office for National Statistics and is licensed under the Open Government Licence v.3.0. Contains OS data © Crown copyright and database right [2024].

The 124 reviewed documents span multiple transport modes (road, railway, aviation, maritime, and active travel) and various geographical scales, from individual towns and small local authorities to larger county councils, combined authorities, and regional transport organisations (e.g. English and Scottish sub–national bodies and regional transport partnerships). Beyond these, the review includes documents pertaining to the individual nations of the UK as well as the country as a whole. Figure 9 summarises the geographical coverage of the reviewed documents, with a full list provided in Table S1. The findings from the document review are presented in the Results section.

### Stakeholder workshops

Two workshops were conducted that collectively brought together over 100 transport policymakers, practitioners, and other key actors to identify challenges and opportunities for decarbonisation, building resilience, and integrating technology and climate science into the UK’s transport strategies. The first workshop was primarily scoping in nature, helping to set the thematic focus for the research and to ensure that the most relevant strategy documents were considered within the review. The second workshop was analytical and synthesis-oriented, beginning with short plenary presentations summarising key findings from the document review, followed by structured breakout group discussions and plenary feedback sessions. Participants were allocated to mixed groups comprising representatives from academia, national and local government, and industry, in order to facilitate cross-sectoral dialogue. The workshop outputs were categorised thematically, distinguishing insights at national, regional, and local scales, which informed the analysis presented in Views from stakeholders.

## Supplementary information

Supplementary Information

## Data Availability

No datasets were generated or analysed during the current study.
