# Supplementary material for: Opportunities and challenges for decarbonised, adaptable and resilient transport systems in the United Kingdom
Source: NPJ Sustain Mobil Transp. 2026 Nov 2;3(1):64. doi: 10.1038/s44333-026-00131-2 (PMC13727689; doi:10.1038/s44333-026-00131-2)
Supplement: Supplementary file 1 [file 44333_2026_131_MOESM1_ESM.pdf]

Table S1 Reviewed strategy documents

Organisation	Title	Number
**National Strategies**		
Civil Aviation Authority	Climate Change Adaptation Report	116
Civil Aviation Authority	Operating Resilience of the UK's aviation infrastructure and the consumer interest	117
Department for Business, Energy & Industrial Strategy	Electricity Networks Strategic Framework: Enabling a secure, net zero energy system	13
Department for Environment, Food and Rural Affairs	UK Climate Change Risk Assessment 2022	21
Department for Environment, Food and Rural Affairs	The Third National Adaptation Programme (NAP3) and the Fourth Strategy for Climate Adaptation Reporting	118
Department for Infrastructure	Ensuring a Sustainable Transport Future: a New Approach to Regional Transportation	120
Department for Infrastructure	DfI Strategic Framework	121
Department for Regional Development	Regional Development Strategy	122
Northern Ireland Assembly	Decarbonising Transport in Northern Ireland	123
Department for Transport	Decarbonising transport - A Better, Greener Britain	1
Department for Transport	Future of Transport: Helping local authorities to unlock the benefits of technology & innovation in rural transport	5
Department for Transport	Future of transport regulatory review: future of flight	6
Department for Transport	Flightpath to the future: a strategic framework for the aviation sector	7
Department for Transport	Future of transport regulatory review: maritime autonomy and remote operations	8
Department for Transport	Clean Maritime Plan	9
Department for Transport	Maritime 2050: Navigating the Future	10
Department for Transport	Future of Transport Regulatory Review: zero emission vehicles	11
Department for Transport	Future of mobility: urban strategy	12
Department for Transport	National Bus Strategy for England: Bus Back Better	47
Department for Transport	Transport Infrastructure Efficiency Strategy	119
Innovate UK / UKRI	UK Transport Vision 2050: Investing in the future of mobility	2
Institution of Civil Engineers	Low Carbon Concrete Routemap: Setting the agenda for a path to net zero	14
Institution of Civil Engineers	State of the Nation 2020: infrastructure and the 2050 net zero target	17
Institution of Civil Engineers	State of the Nation 2022: Improving infrastructure productivity	18
Mineral Products Association	UK Concrete and Cement Industry Roadmap to Beyond Net Zero	15

Table S1 Continued: Reviewed strategy documents.

Organisation	Title	Number
**National Strategies - Continued**		
National Infrastructure Commission	National Infrastructure Assessment	22
National Infrastructure Commission	Delivering net zero, climate resilience and growth	23
National Infrastructure Commission	Infrastructure Progress Review 2023	24
National Preparedness Commission	Building Confidence in the Future: Towards a framework for measuring national resilience	29
Northern Lighthouse Board	Our Journey to Sustainability	114
The Transport Research and Innovation Board	TRIB - digital twin Roadmap 2035	28
Trinity House	Climate Change Adaptation Report: Third round	115
UK Government Infrastructure and Projects Authority	Transforming Infrastructure Performance: Roadmap to 2030	25
UK Green Building Council	Net Zero Whole Life Carbon Roadmap: A Pathway to Net Zero for the UK Built Environment	16
National Grid	Grid Guide to the Decarbonisation of Transport	26
Network Rail	Network Rail Environmental Sustainability Strategy 2020-2050	3
Network Rail	Weather Resilience and Climate Change Adaptation plans (Network and Route level)	20
Active Travel England	Active Travel England Corporate Plan 2023-2025	113
National Highways	Net zero highways: our 2030 / 2040 / 2050 plan	4
Planning Advisory Service	Joined Up Thinking in Uncertain Times: Links between Strategic Planning and Transport	19
Royal Town Planning Institute	Net Zero Transport: The role of spatial planning and place-based solutions	27
Scottish Government	Climate Ready Scotland: climate change adaptation programme 2019-2024	30
Scottish Government	Mission Zero for Transport	97
Transport Scotland	National Transport Strategy 2	52
Welsh Government	National transport delivery plan 2022 to 2027	31
Welsh Government	Llwybr Newydd: the Wales transport strategy 2021	98
Welsh Government	Transport: Sector Emission Pathway	99
Welsh Government	Decarbonisation of Transport	124
**Regional Strategies**		
Shetland Transport Partnership (ZetTrans)	Shetland Transport Strategy 2018-2028	105
Shetland Transport Partnership (ZetTrans)	Shetland Transport Strategy 2022-2042	106
Highlands and Islands Transport Partnership (HITRANS)	2008 Regional Transport Strategy	101

Table S1 Continued: Reviewed strategy documents.

Organisation	Title	Number
**Regional Strategies - Continued**		
North-East of Scotland Transport Partnership (NESTRANS)	Regional Transport Strategy For The North East Of Scotland	103
Strathclyde Partnership for Transport (SPT)	A Call to Action: The Regional Transport Strategy for the west of Scotland 2023-2038	109
Tayside and Central Scotland Transport Partnership (TACTRAN)	Tayside and Central Scotland Regional Transport Strategy 2024-2034	110
South-West of Scotland Transport Partnership (Swestrans)	SWestrans Regional Transport Strategy 2023-42	108
South-East of Scotland Transport Partnership (SESTRAN)	SEStran 2035 Regional Transport Strategy	107
Transport for the North	Transport Decarbonisation Strategy	43
Midlands Connect	Fairer, greener, stronger: A Strategic Transport Plan for the Midlands	102
Western Gateway	Western Gateway Strategic Transport Plan 2024-2050	112
Transport East	Transport East Transport Strategy 2023-2050	111
Transport for London	Mayor's Transport Strategy	33
Peninsula Transport	Carbon Transition, Rural mobility and Freight Strategy for the South West	104
England's Economic Heartland	Transport Strategy: Connecting People, Transforming Journeys	100
Transport for the South East	Transport Strategy for the South East	32
**Combined Local Authority Strategies**		
North East Combined Authority	North East Transport Plan 2021-2035	53
Tees Valley Combined Authority	Strategic Transport Plan	96
West Yorkshire Combined Authority	Transport Strategy 2040	35
Greater Manchester Combined Authority	Greater Manchester Transport Strategy 2040	79
Liverpool City Region Combined Authority	Liverpool City Region Combined Authority Transport Policies	94
South Yorkshire Combined Authority	Transport Strategy and Vision for South Yorkshire	95
Transport for West Midlands	Reimagining transport in the West Midlands	54
Transport for West Midlands	Transport Network Resilience Plan	55
West of England Combined Authority	Transport Strategy for West of England	40

Table S1 Continued: Reviewed strategy documents.

Organisation	Title	Number
**Local Authority Strategies**		
Aberdeenshire County Council	Local Transport Strategy 2023 for Aberdeen	62
Barnet London Borough Council	Barnet Long Term Transport Strategy 2020-2041	84
Basingstoke City Council	Basingstoke Transport Strategy	39
Birmingham City Council	Birmingham Transport Plan	42
Bracknell Forest Council	Local Transport Plan 3 for Bracknell Forest Council	75
Brent Council	Transport Strategy for Brent Council: Long Term 2015-2035	36
Brent Council	Transport Strategy for Brent Council: Cycle Strategy 2016-2021	37
Brent Council	Transport Strategy for Brent Council: Walking Strategy 2017-2022	38
Bristol City Council	Bristol Transportation Strategy	34
Calderdale Council	Calderdale Transport Strategy 2016-2031	80
Cambridgeshire County Council	Cambridgeshire County Council	63
Central Bedfordshire Council	Transport Strategy for Central Bedfordshire	41
City of Edinburgh Council	Edinburgh City Mobility Plan	89
City of Stoke-on-Trent	Transport Strategy and Delivery Plan for City of Stoke-on-Trent	48
Cornwall Council	Local Transport Plan for Cornwall Council	44
Coventry City Council	Transport Strategy for Coventry	45
Derbyshire County Council	Carbon reduction Strategy Road Transport Network for Derbyshire	51
Devon County Council	Devon Local Transport Plan 4 (2026-40)	83
East Dunbartonshire Council	Active Travel Strategy 2023-30: Evidence Summary and Approach for East Dunbartonshire	70
East Dunbartonshire Council	Local Transport Strategy for East Dunbartonshire	71
East Renfrewshire Council	Local Transport Strategy for East Renfrewshire (Case for Change)	67
East Riding of Yorkshire Council	Local Transport Plan for East Riding + Appendix B Network Resilience Plan	50
East Sussex County Council	Local Transport Plan for East Sussex	90
Glasgow City Council	Glasgow Transport Strategy	73
Hampshire County Council	Local Transport Plan for Hampshire	60
Herefordshire Council	Local Transport Plan (2016-31) for Herefordshire - Strategy Document	88
Hertfordshire Council	Local Transport Plan (2018-2031) for Hertfordshire	92
Islington Council	Transport Strategy 2019-41 for Islington	87
Kent County Council	Emerging Local Transport Plan for Kent: Turning The Curve Towards Net Zero	81
Lambeth Council	Transport Strategy for Lambeth	68
Leeds City Council	Connecting Leeds Transport Strategy	46
Leicester City Council	Local Transport Plan for Leicester City Council	91

Table S1 Continued: Reviewed strategy documents.

Organisation	Title	Number
**Local Authority Strategies - Continued**		
London Borough of Harrow	Harrow transport policy documents (road saftety, walking, cycling and sustainable transport)	76
Manchester City Council	Transport Strategy for Manchester	57
North Ayrshire Council	Transport Strategy for North Ayrshire	72
North Lincolnshire Council	Local Transport Plan 2011-2026	86
Oxfordshire County Council	Local Transport and Connectivity Plan for Oxfordshire	78
Plymouth City Council	Transport Plans for Plymouth	56
Portsmouth City Council	Transport Strategy for Portsmouth	61
Reading Borough Council	Reading Transport Strategy 2040	77
Sheffield City Council	Sheffield's Transport Strategy (2019-35)	93
Solihull Metropolitan Borough Council	Solihull Connected	74
Somerset County Council	Somerset's Future Transport Plan	65
South Lanarkshire Council	Local Transport Strategy 2024-34 for South Lanarkshire	82
Southampton City Council	Connected Southampton 2040	64
Staffordshire County Council	Local Transport Plan for Staffordshire	69
Suffolk County Council	Suffolk's Local Transport Plan 2011-2031 Part 1 - Transport Strategy & Part 2 - Implementation Plan	49
Surrey County Council	Local Transport Plan for Surrey	59
Sutton Council	Sustainable Transport Strategy for Sutton	58
Warwickshire County Council	Local Transport Plan (LTP4) for Warwickshire (Draft)	85
West Sussex County Council	West Sussex Transport Plan	66

Table S2 Carbon Emission Reduction Strategies by Industry

Code	Carbon Emissions Reduction Strategies	Industry
A1	Low carbon fuels in road and rail transportation	Transport
A2	Low temperature asphalt	Asphalt Industry
A3	Reuse/Circular economy	Asphalt Industry
A4	Alternative fuels (Biomass/Hydrogen)	Fuel Supply & Hydrogen
A5	Process efficiencies/waste reduction	Asphalt Industry
A6	Alternative fuels (Biomass/Hydrogen)	Fuel Supply & Hydrogen
C1	Grid decarbonisation	Power & Energy
C2	Low carbon fuels in road and rail transportation	Transport
C3	Low carbon fuels in road and rail transportation	Transport
C4	Design/construction efficiency	Construction Industry
C5	Mix design/formulation (GGBS/Flyash/Limestone/Admixtures)	Concrete Industry
C6	Mix design/formulation (GGBS/Flyash/Limestone/Admixtures)	Construction Industry
C7	Mix design/formulation (GGBS/Flyash/Limestone/Admixtures)	Concrete Industry
C8	Mix design/formulation (GGBS/Flyash/Limestone/Admixtures)	Construction Industry
C9	Design/construction efficiency	Construction Industry
C10	Design/construction efficiency	Construction Industry
C11	Process efficiencies/waste reduction	Construction Industry
C12	Design/construction efficiency	Construction Industry
C13	Design/construction efficiency	Construction Industry
C14	Design/construction efficiency	Construction Industry
C15	Process efficiencies/waste reduction	Concrete Industry
C16	Mix design/formulation (GGBS/Flyash/Limestone/Admixtures)	Concrete Industry
C17	Process efficiencies/waste reduction	Concrete Industry
C18	Carbon capture and utilisation/storage	Green-house Gas Removals
C19	Grid decarbonisation	Power & Energy
C20	Design/construction efficiency	Construction Industry
C21	Grid decarbonisation	Power & Energy
C22	Low carbon fuels in road and rail transportation	Transport
C23	Mix design/formulation (GGBS/Flyash/Limestone/Admixtures)	Concrete Industry
C24	Alternative fuels (Biomass/Hydrogen)	Fuel Supply & Hydrogen
C25	Carbon capture and utilisation/storage	Green-house Gas Removals
C26	Mix design/formulation (GGBS/Flyash/Limestone/Admixtures)	Concrete Industry
C27	Mix design/formulation (GGBS/Flyash/Limestone/Admixtures)	Construction Industry
C28	Mix design/formulation (GGBS/Flyash/Limestone/Admixtures)	Concrete Industry
C29	Mix design/formulation (GGBS/Flyash/Limestone/Admixtures)	Construction Industry
C30	Process efficiencies/waste reduction	Concrete Industry
C31	Carbon capture and utilisation/storage	Green-house Gas Removals
C32	Carbon capture and utilisation/storage	Green-house Gas Removals
C33	Mix design/formulation (GGBS/Flyash/Limestone/Admixtures)	Concrete Industry
C34	Alternative fuels (Biomass/Hydrogen)	Fuel Supply & Hydrogen
C35	Grid decarbonisation	Power
C36	Grid decarbonisation	Power
C37	Alternative fuels (Biomass/Hydrogen)	Fuel Supply & Hydrogen
C38	Grid decarbonisation	Power
C39	Low carbon fuels in road and rail transportation	Transport

Table S2 Continued: Carbon Emission Reduction Strategies by Industry

Code	Carbon Emissions Reduction Strategies	Industry
C40	Mix design/formulation (GGBS/Flyash/Limestone/Admixtures)	Concrete Industry
C41	Alternative fuels (Biomass/Hydrogen)	Fuel Supply & Hydrogen
C42	Grid decarbonisation	Power
C43	Carbon capture and utilisation/storage	Green-house Gas Removals
S1	Low carbon fuels in road and rail transportation	Transport
S2	EAF steelmaking	Steel Industry
S3	Alternative fuels (Biomass/Hydrogen)	Fuel Supply & Hydrogen
S4	Carbon capture and utilisation/storage	Green-house Gas Removals
S5	Process efficiencies/waste reduction	Steel Industry
S6	Design/construction efficiency	Construction Industry
S7	Design/construction efficiency	Construction Industry
S8	Reuse/Circular economy	Steel Industry
S9	Design/construction efficiency	Construction Industry
S10	Reuse/Circular economy	Steel Industry
S11	Process efficiencies/waste reduction	Steel Industry
S12	Alternative fuels (Biomass/Hydrogen)	Fuel Supply & Hydrogen
S13	Grid decarbonisation	Power & Energy
S14	Carbon capture and utilisation/storage	Green-house Gas Removals
S15	Low carbon fuels in road and rail transportation	Transport
S16	Design/construction efficiency	Construction Industry
S17	Process efficiencies/waste reduction	Steel Industry
S18	Reuse/Circular economy	Steel Industry
S19	EAF steel-making	Steel Industry
S20	Carbon capture and utilisation/storage	Green-house Gas Removals
S21	Grid decarbonisation	Power & Energy

Table S3 Framework segments with their associated analytical components ('fingers') and descriptions

Segment	Analytical Component	Description
Risk assessment	Integrating updated climate science	Applying the latest climate models and projections to anticipate risks and inform adaptation strategies.
	System thinking and criticality assessment	Identifying critical assets and interdependencies across infrastructure systems to evaluate vulnerabilities.
	Uncertainty and sensitivity to future changes	Testing robustness of strategies under a range of possible future conditions and assumptions.
	Comprehensive impact analysis	Assessing social, economic, environmental, and safety consequences of disruptions or interventions.
Sustainability	Social impact	Ensuring equitable access, inclusion, and wider social wellbeing in transport planning.
	Biodiversity impact	Evaluating effects of infrastructure on habitats, ecosystems, and ecological resilience.
	Pollution and carbon emissions	Accounting for embodied and operational carbon, as well as air, noise, and other pollutants.
	Resource efficiency	Promoting efficient use of materials, energy, and land, including circular economy approaches.
Condition assessment	Monitoring to improve understanding	Using inspections, sensors, and data systems to evaluate the current state of assets.
	Condition projection	Forecasting deterioration and performance over time under changing loads and hazards.
	Extreme loading (incl. climate)	Assessing how assets perform under compound stresses, including extreme weather (e.g., flooding, heatwaves, storms) and traffic-related loads (e.g., HGV surges, overloading, peak travel demands).
	Unified metrics for asset inter-comparison	Developing standardised measures to compare risks and conditions across different asset types.
Resilience	Resilience standards / metrics	Establishing benchmarks and indicators for resilience performance across the sector.
	Interdependencies between and within systems	Considering cross-sectoral (e.g., transport–energy) and intra-sectoral links (e.g., road-drainage) to avoid cascading failures.
	Recovery and response planning	Preparing contingency and emergency response measures to restore service after disruptions.
	Service continuity	Designing redundancy and alternative pathways to maintain essential functions during shocks.
Travel demand insights	Transport demand modelling	Capturing current and projected travel patterns across modes, purposes, and geographies.
	Land use and transport interaction	Linking settlement patterns and spatial development to transport demand and capacity.
	Travel behaviour	Understanding user choices, equity issues, and how preferences evolve over time.
	Transport planning and scheduling	Evaluating options for service operations, timetabling, and associated resource allocation to align with demand.
